# Multi-Camera Analysis of Simulated Porcine Recovery States Based on Three-Dimensional Pose Using Synthetic Data

**DOI:** 10.3390/jimaging12090429

**Published:** 2026-09-10

**Authors:** Artem Obukhov, Daniil Teselkin, Maxim Shiltsyn, Denis Dedov, Marina Nikitina, Liliya Fedulova, Irina Chernukha

**Affiliations:** 1 Laboratory of VR Simulators, Tambov State Technical University, Tambov 392000, Russia; dteselk@mail.ru (D.T.); maks.shilcin@mail.ru (M.S.); hammer68@mail.ru (D.D.); 2 V. M. Gorbatov Federal Research Center for Food Systems, Moscow 109316, Russia; m.nikitina@fncps.ru (M.N.); l.fedulova@fncps.ru (L.F.); i.chernuha@fncps.ru (I.C.)

**Keywords:** computer vision, animal pose estimation, synthetic data, domain randomization, multi-camera reconstruction, three-dimensional keypoints, behavioral monitoring, temporal state classification

## Abstract

Continuous objective assessment of pig motor condition, including recovery after anesthesia, requires the joint analysis of posture, locomotion, transitions between states, and short-term adverse events. This study aimed to develop and algorithmically evaluate a multi-camera pipeline for the quantitative analysis of simulated recovery states in a fully synthetic virtual environment. Procedural generation was used to produce 150,000 images annotated with fifteen anatomical keypoints while varying the pose, size, and appearance of the model, camera viewpoints, illumination, environment, and image post-processing parameters. The YOLO11m-pose model was used for two-dimensional pose estimation, after which observations from three synchronized cameras were combined using weighted triangulation. The reconstructed three-dimensional trajectories were processed using a quality-control system, a finite-state machine, and temporal rules for detecting falls, prolonged immobility, and convulsion-like movements. After repartitioning the dataset by three-dimensional pose index, the retrained YOLO11m-pose model achieved a keypoint ${\mathrm{mAP}}_{50–95}$ of 0.8968, a precision of 0.9985, and a recall of 0.9986 on the independent pose-level test set. During the processing of a 15-min three-camera sequence, 98.993% of 405,000 reconstructions satisfied the geometric acceptance criteria, and the median reprojection error was 2.420 pixels. On a first manually annotated 5-min synthetic sequence, the state machine achieved a strict frame-level accuracy of 91.20%, a macro-F1 score of 90.10%, and Cohen’s κ of 0.860; accuracy outside ±1 s neighborhoods of state transitions was 97.43%. On a second 5-min synthetic evaluation sequence with exact Unity-world geometric ground truth, direct three-dimensional evaluation yielded an MPJPE of 31.86 mm at 99.993% landmark coverage. A limited temporal assessment on this second sequence detected both of the two lying-derived prolonged-immobility reference intervals; fall and convulsion-like-movement events were not independently annotated. All training, validation, and end-to-end evaluation data were generated in a virtual environment; therefore, these results establish algorithmic feasibility within the synthetic domain but do not establish performance on real animals.

## 1. Introduction

Continuous monitoring of pig condition is required in a range of husbandry and veterinary situations, including disease, farrowing, and recovery after procedures involving anesthesia. In such cases, objective assessment requires monitoring not a single sign but the dynamics of interrelated motor manifestations. These include the time required to assume a stable standing posture, the ability to perform purposeful locomotion, the duration of recumbency, the frequency of unsuccessful attempts to stand, falls, prolonged immobility, and abnormal repetitive movements. These signs have different clinical significance and change over time. In particular, a reduction in recovery time after anesthesia does not necessarily indicate improved animal welfare if it is accompanied by struggling during attempts to stand, falls, or other adverse reactions [1]. Behavioral signs are also used in the assessment of pain in pigs, and the contributions of individual postures and movement patterns to the final assessment are not equivalent [2]. Therefore, the monitored condition should be described not by a single “normal/pathological” label but by a temporal profile representing the proportions of favorable, neutral, unfavorable, and uncertain states. In the present study, recovery after anesthesia is considered a representative monitoring scenario, whereas the computational pipeline is intended for broader long-term monitoring of pig motor condition.

Traditional observation is performed periodically by a specialist and is usually documented using a scale or protocol. This approach remains clinically relevant; however, it depends on the observer’s experience, is limited by the available observation time, and may fail to capture short-term events occurring between assessment points. Similar limitations have been reported in studies on the automatic recognition of pain in animals: manual assessment requires expert training, is subject to inter-observer variability, and complicates continuous monitoring [3]. Video analytics does not replace clinical decision making, but it can transform a continuous recording into a reproducible sequence of measurements while preserving the possibility of subsequent expert verification of each detected event.

Modern computer vision systems used in pig farming are already capable of detecting animals in video frames, distinguishing basic postures, and tracking their behavior. Recent studies have demonstrated long-term quantification of the durations of standing, lying, feeding, and other activities using combinations of object detectors and tracking algorithms [4,5], posture recognition under group-housing conditions [6], and body-posture detection under different illumination conditions [7]. These results confirm the feasibility of non-invasive quantitative monitoring. However, most such solutions generate two-dimensional bounding boxes or discrete class labels. This representation is insufficient for a detailed analysis of simulated recovery states: the same recumbent posture may correspond to a calm state, a predefined phase of emergence, or a critical event, whereas the same two-dimensional projection may result from different spatial configurations of the body.

A more informative representation of movement is provided by pose estimation based on anatomical keypoints. The coordinates of the head, trunk, and limbs make it possible to calculate body orientation, the heights of individual segments, skeletal configuration, the velocity of the geometric center of body mass, and gait characteristics. A study of sow locomotion demonstrated the applicability of markerless keypoint detection for subsequent kinematic analysis [8], whereas GANPose and MMVSL demonstrated the progression from pose recognition toward the analysis of skeletal topology and pig actions in complex scenes [9,10]. Nevertheless, two-dimensional pose estimation remains dependent on viewpoint and occlusions; consequently, a change in camera position may imitate a change in the animal’s state.

The use of several synchronized cameras makes it possible to reconstruct the spatial coordinates of keypoints through triangulation and to distinguish animal movement from perspective distortion. For a software system, this introduces an additional requirement: interpretation of the simulated state should be performed only after the technical quality of the reconstruction has been evaluated. Low detector confidence, the absence of a keypoint in several projections, a large reprojection residual, or a violation of the expected skeletal proportions do not represent critical events within the scenario. Instead, they should assign the observation to a category of insufficient reliability; otherwise, a technical failure may be interpreted as a false event.

The main limitation in training pose-estimation models is the cost of obtaining representative annotations. For each frame, anatomical keypoints must be marked manually, including points that are occluded by the animal’s body or by elements of the environment. Real examples of rare, short-term, and potentially hazardous scenarios—including falls, convulsion-like movements, unnatural postures, and prolonged immobility episodes—are especially scarce. The deliberate acquisition of such data from animals is ethically unacceptable, whereas naturally collected datasets are generally imbalanced. Consequently, high accuracy for common postures does not guarantee sensitivity to the events that are most important for continuous condition monitoring.

Synthetic data make it possible to obtain images and accurate annotations simultaneously, control the frequency of rare scenarios, and systematically vary the imaging conditions. Contemporary reviews associate their use with reductions in annotation workload, class imbalance, and sampling bias [11]. The Animal3D study confirmed the value of large-scale annotated representations for three-dimensional animal pose estimation [12], whereas more recent studies have demonstrated the possibility of using geometric constraints and reprojection to train three-dimensional pose-estimation models when direct three-dimensional annotations are scarce [13]. In a fully synthetic study, variability in model size and appearance, pose, viewpoint, illumination, and environment is intended to reduce dependence on incidental features of a particular virtual configuration; however, such randomization alone does not establish generalization beyond the generated synthetic domain.

For this purpose, the present study developed a synthetic virtual observation environment and a parametric pig model. Procedural generation covered the animal’s position, orientation, and size; animation states; textures and material parameters; illumination; environment; virtual-camera parameters; and image post-processing. For each pose configuration, twelve visually distinct projections were generated. To prevent projections of the same underlying three-dimensional state from appearing in different data subsets, all projections sharing the same pose index were subsequently assigned to the same training, validation, or test subset. The synthetic dataset contained 150,000 images annotated with fifteen anatomical keypoints and was used to train a YOLO Pose model entirely within the virtual domain.

The trained model was integrated into a complete multi-camera software pipeline. Three synchronized video sequences generated in the virtual scene were processed frame by frame. The two-dimensional keypoints underwent filtering, matching, and three-dimensional triangulation, after which the trajectory of the geometric center of body mass, traveled distance, velocity, posture, transitions between postures, prolonged immobility episodes, falls, and convulsion-like movements were calculated. The results were temporally aggregated into proportions of the author-defined categories of simulated recovery. A recumbent posture was not automatically classified as unfavorable: its interpretation depended on the movement dynamics, duration, preceding transition, detected critical events, and observation quality.

The research question was formulated as follows: can a keypoint model trained on procedurally generated images, together with multi-camera reconstruction and temporal rules, provide a reproducible quantitative assessment of the dynamics of predefined recovery scenarios in a synthetic virtual environment? The testable hypothesis was that broad procedural variability within the synthetic domain and an explicit separation between measurement quality and the simulated functional state would enable analysis of new synthetic video sequences within the predefined virtual domain and allow the results to be represented as the percentage of time assigned to favorable, neutral, unfavorable, and uncertain categories, together with the temporal localization of critical events.

The aim of this study was to develop and algorithmically evaluate, within a virtual environment, a multi-camera video-analysis method for the quantitative description of predefined pig recovery scenarios based on keypoints extracted by a neural network trained on a synthetic dataset. To achieve this aim, four interrelated objectives were addressed: (1) the procedural generation of a large-scale synthetic image dataset with controlled variability and automatic annotation; (2) the training and evaluation of a model for object detection and anatomical keypoint localization; (3) the reconstruction of spatial pose and the calculation of motor and biomechanical indicators using three virtual cameras; and (4) the development of an author-defined temporal classification of the simulated state, with separate consideration of observation quality and critical events.

The principal contribution of this study is the integration of components that have previously been considered separately into a single reproducible pipeline. The synthetic virtual environment and procedural randomization are used to train object detection and anatomical pose estimation; multiple cameras transform two-dimensional predictions into spatial measurements; quality control prevents computer vision errors from being conflated with critical states within the scenario; and the final analytics describe simulated recovery through temporal proportions and events that can be verified using the system logs. The categories are generated by the developed classifier and are not intended to provide a clinical interpretation.

The remainder of the article follows an extended IMRAD structure. Section 2 reviews contemporary methods for pig behavior recognition, pose estimation, the use of synthetic data, and multi-camera analysis. Section 3 describes the synthetic virtual environment, procedural-randomization parameters, dataset generation, YOLO Pose training, multi-camera geometry, triangulation, and the algorithm for classifying simulated recovery states. Section 4 presents the results of model training and synthetic-video processing. Section 5 discusses the scientific and software-related interpretation of the results, the limitations of the fully virtual experiment, and the boundaries of applicability of the conclusions. Section 6 summarizes the principal findings of the study.

## 2. Related Work

### 2.1. Computer Vision and Quantitative Monitoring of Pig Behavior

The development of precision livestock farming has led to a transition from episodic observation to the automated analysis of continuous video. For pigs, the most common tasks include individual animal detection, posture or action recognition, and the preservation of animal identity over time. Tu et al. combined YOLOv5 and ByteTrack and demonstrated the feasibility of calculating the duration of individual behavioral states [4]. In a subsequent study, a combination of YOLOv8 and OC-SORT was used for the long-term monitoring of group behavior under occlusions, changing illumination, and mutual overlap between animals [5]. These approaches are methodologically important because they transform sequences of frame-level classifications into time-budget indicators.

Posture recognition from RGB images remains widely used because of the availability of conventional cameras. Devi et al. applied instance segmentation to distinguish between sternal and lateral recumbency, walking, and sitting in group scenes [6]. Scaillierez et al. investigated posture and location detection under different illumination conditions and demonstrated that the imaging conditions affect both model performance and the observed behavior [7]. For a synthetic dataset, this implies that illumination and image-formation parameters should be considered substantial sources of variability rather than merely cosmetic properties of the scene.

An alternative to direct frame-by-frame classification is the analysis of state sequences. Mluba et al. used behavioral pattern mining to monitor pig health and welfare [14]. This approach corresponds to a temporal analysis framework in which not only the class assigned to the current frame but also the order, duration, and recurrence of states are significant. For example, short-term recumbency, prolonged absence of movement, and repeated transitions between lying and standing have different meanings, although they may contain visually similar frames.

When changes in functional condition are monitored, recovery from anesthesia represents an illustrative case in which an expected phase of emergence must be distinguished from an adverse reaction. Layton et al. evaluated not only the time required for pigs to recover from anesthesia but also recovery characteristics and undesirable behavioral manifestations [1]. Trindade et al. demonstrated that pain-related behavioral signs have unequal statistical weights [2]. A review of animal pain-recognition technologies also emphasized the limitations of subjective manual assessment and the need for reliable ground-truth annotations [3]. It follows that a simple cumulative percentage of activity is not a sufficient measure: an algorithmic recovery profile should preserve the structure of the original indicators and enable verification of the reasons for assigning a particular interval to a given category.

The systems considered above are primarily intended for farms and group-housing conditions, whereas detailed condition monitoring often concerns an individual animal and requires a more comprehensive assessment of its kinematics. A two-dimensional detector effectively answers the questions “Where is the animal located?” and “What general posture has it assumed?” but provides a less detailed description of stability, symmetry, micromovements, and spatial trajectory. Therefore, recovery assessment requires a transition from object bounding boxes and action classes to anatomical keypoints, three-dimensional geometry, and temporal features.

### 2.2. Pose Estimation, Synthetic Data, and Multi-Camera Reconstruction

Markerless pose estimation represents an animal as a set of anatomical landmarks and connections between them. de Paula et al. developed a video repository of sows with different locomotion scores and compared skeletal configurations and keypoint-detection architectures [8]. Their study demonstrated that the selection of landmarks should be determined by the subsequent measurement task: limb keypoints are required for gait analysis, whereas a sparser skeleton may be sufficient for coarse posture classification. In the present study, fifteen keypoints were selected as a compromise between detection robustness and the ability to evaluate the positions of the head, trunk, and weight-bearing segments.

Occlusion is the principal obstacle in group scenes. GANPose uses generative adversarial learning to reconstruct the keypoints of overlapping pigs [10]. MMVSL combines global image features, local pose features, and skeletal topology, thereby supporting both pose estimation and action recognition [9]. These studies confirm that structural relationships between keypoints contain information that is absent from independent coordinates. Nevertheless, they predominantly evaluate the quality of two-dimensional pose estimation or action classification and do not fully address the problem of metric spatial reconstruction using multiple cameras.

Three-dimensional animal pose estimation is complicated by data scarcity and perspective ambiguity. The Animal3D dataset systematized three-dimensional animal poses and shapes and demonstrated the importance of geometrically consistent annotations for model training [12]. Dai et al. proposed a geometric self-supervision approach in which a predicted three-dimensional pose is reprojected into random two-dimensional viewpoints, thereby forming consistency constraints [13]. The general concept underlying these studies is consistent with the motivation for a multi-camera approach: a single viewpoint often permits several spatial interpretations, whereas additional projections or geometric constraints reduce this ambiguity.

Synthetic data make it possible to obtain accurate keypoints, bounding boxes, and camera parameters without manual annotation. The review by Delussu et al. highlighted the advantages of controlled dataset generation, balancing of rare cases, and scalable annotation [11,15]. In a fully virtual study, a key requirement is the diversity of the synthetic distribution. If all frames contain the same background, illumination, and viewpoint, the model may rely on persistent features of a particular scene instead of the shape and configuration of the animal.

Procedural randomization expands the distribution of synthetic observations. In the developed generator, the animal’s position and orientation, size, animation, textures and material parameters, environment configuration, intensity and color of light sources, field of view, exposure, contrast, saturation, vignetting, grain, and chromatic aberration were varied independently. Twelve fixed camera positions generated different projections of the same parametric model. Because a viewpoint change substantially modifies the relative arrangement of keypoints, perspective, visibility, and self-occlusion for a two-dimensional neural network, each projection was treated as a separate pose sample. This approach increases the diversity of observation geometries and enables the controlled generation of rare scenarios. Research on the development of digital technologies in pig farming considers computer vision, automated monitoring, and digital models to be complementary elements of an intelligent system [16], whereas systematic analyses of public datasets emphasize the need for controlled variability, complete annotations, and transparent metadata descriptions [17].

In a fully virtual study, at least three levels of algorithmic evaluation should be distinguished: model performance on a synthetic validation dataset, the geometric consistency of two-dimensional keypoints across virtual cameras, and the stability of the derived spatiotemporal indicators. A high mAP value characterizes the model’s ability to learn the generated synthetic distribution, but does not by itself confirm the consistency of the three-dimensional reconstruction or the temporal classifier. Therefore, predictions are additionally evaluated across cameras and over time, and intervals with insufficient reliability are not automatically assigned to unfavorable states. Contemporary reviews of visual pig-monitoring technologies also indicate that state interpretation requires the joint analysis of appearance, posture, movement, and physiological microfeatures rather than a single frame-level label [18,19].

### 2.3. Research Gap and Contribution of the Present Study

An analysis of publications from 2022 to 2025 indicates that the required components are developing rapidly but are generally investigated separately. Behavioral-monitoring studies effectively address detection, tracking, and the calculation of the durations of several behavioral classes [4,5,14]; pose-estimation studies improve anatomical localization accuracy and robustness to occlusions [8,9,10]; and three-dimensional and synthetic datasets advance geometric representations and reduce dependence on manual annotation [11,12,13]. However, an end-to-end framework that connects synthetic training of pig pose estimation, multi-camera three-dimensional reconstruction, measurement-quality control, detection of critical temporal events, and a final author-defined classification of simulated recovery expressed as percentages of observation time remains insufficiently investigated. Studies on contactless morphometry further confirm that semantically defined anatomical landmarks can serve as a basis for calculating body dimensions and spatial characteristics [20,21].

The methodological foundation of animal pose estimation has also expanded considerably. A review of contemporary methods systematized two-dimensional and three-dimensional problem formulations, datasets, and performance metrics [22]. SLEAP implements multi-animal pose estimation and temporal tracking [23]; SuperAnimal uses pretrained cross-species models [24]; and Lightning Pose improves the temporal stability of predictions through semi-supervised learning and Bayesian ensembling [25]. Geometric prior constraints have been applied to improve the consistency of anatomical keypoints [26]. For pigs, both RGB-image-based posture-classification systems [27] and multi-task models that jointly perform keypoint localization, segmentation, and posture estimation [28] are being developed.

The first research gap concerns rare states. Available video datasets are naturally biased toward common behaviors, whereas falls, convulsion-like movements, prolonged immobility episodes, and unnatural postures are represented only to a limited extent. A synthetic virtual environment makes it possible to define their frequency safely and controllably. The second gap concerns interpretation: the “lying” class cannot be considered unambiguously unfavorable, and the state should therefore be determined from a combination of posture, movement dynamics, event duration, and context.

The present study addresses these gaps at the level of a unified software and analytical pipeline. Unlike solutions limited to two-dimensional behavior classification, the proposed approach uses a fifteen-keypoint pose representation and three synchronized cameras to obtain spatial and temporal features. Unlike direct binary frame classification, it first generates explainable intermediate indicators—posture, velocity, distance, transitions, prolonged immobility, and other critical events—and only then aggregates them into the proportions of favorable, neutral, unfavorable, and uncertain states. Finally, observation quality is treated as an independent measurement, allowing a critical state within the scenario to be distinguished from insufficient visual data. The transition from semantic landmarks to measurable spatial characteristics has been demonstrated for three-dimensional pig point clouds [29], whereas the use of keypoints for tracking and quantitative activity assessment has been implemented in contemporary piglet-monitoring systems [30]. Long-term extraction of digital behavioral traits from video recordings is also considered an independent source of quantitative animal characteristics [31].

Thus, the present study lies at the intersection of computer vision, synthetic data, and the quantitative analysis of simulated animal behavior. Its novelty is determined not by an individual detector architecture but by the organization of the data and computations: a procedurally randomized synthetic virtual environment enables scalable pose-estimation training; multi-camera geometry transforms predictions into spatial trajectories; and a temporal model transforms these trajectories into a verifiable author-defined assessment of recovery dynamics. All conclusions are formulated within the boundaries of the predefined synthetic domain. Temporal analysis is particularly important for recognizing hazardous and short-term events: sequential models have been applied to behavior recognition and dangerous-event detection in video recordings [32], whereas specialized algorithms have been developed for the automatic detection of undesirable actions in groups of pigs [33]. Contactless extraction of respiratory micromovements from RGB video has been demonstrated for monitoring respiratory rate in pigs [34] and has also been considered in intelligent systems for respiratory health monitoring [35]. Such physiological information represents a potential extension of the present movement-based pipeline rather than an input used by the current implementation.

## 3. Materials and Methods

### 3.1. Study Design and Computational Environment

The study was conducted entirely in the synthetic domain and followed a computational proof-of-concept design. No physical animals, biological samples, or recordings obtained in a vivarium or farm environment were used. Therefore, animal inclusion criteria, randomization of biological groups, and veterinary interventions were not applicable, and approval by a bioethics committee was not required. The terms favorable, neutral, unfavorable, and uncertain state hereafter denote author-defined algorithmic categories of the virtual scenario. They were used to analyze the operation of the software pipeline and do not represent clinical classes.

**Figure 1:**
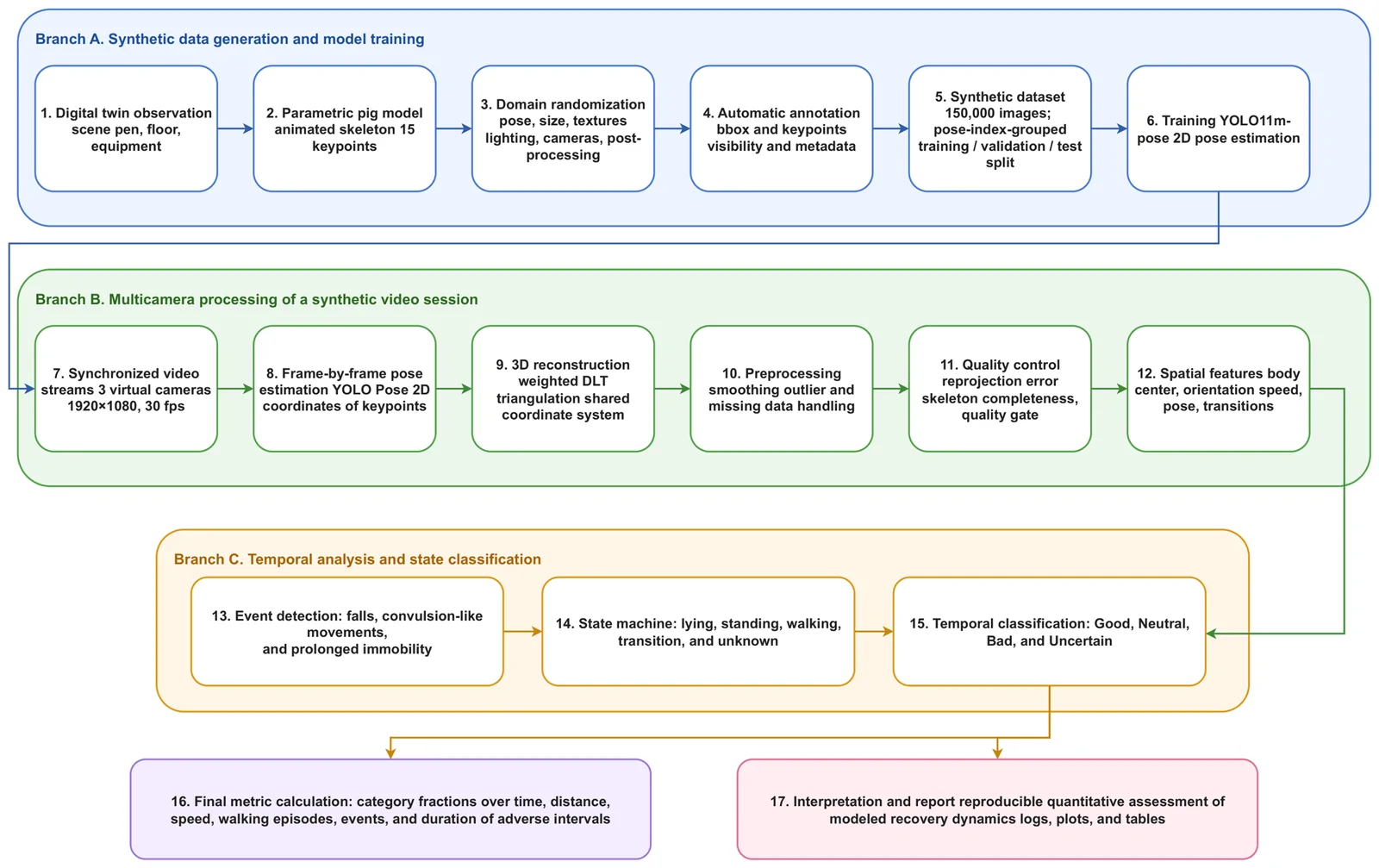
Overall structure of the developed software and analytical pipeline.

The overall structure of the developed pipeline is presented in Figure 1.

The computational experiment comprised two related stages. In the first stage, a procedural generator produced static images of a parametric pig model with accurate automatic annotations of the bounding box and anatomical keypoints; these data were used for training and synthetic validation of the YOLO pose-estimation model. In the second stage, the trained model was applied to three synchronized video streams representing the same subject domain but acquired from separate virtual-camera positions and containing a temporally changing pose. The two-dimensional predictions were combined using geometric triangulation, after which the three-dimensional pose, trajectory, locomotor features, technical quality, and simulated critical events were calculated. A similar separation between two-dimensional pose estimation and multi-camera reconstruction is used in contemporary systems for three-dimensional animal analysis [36,37].

The digital scene was developed in Unity 6000.4.5f1 using Universal Render Pipeline 17.4.0. The neural-network component was implemented in Python 3.12.10 using Ultralytics 8.4.90 and PyTorch 2.4.1+cu124 with CUDA support. Computations were performed on an NVIDIA GeForce RTX 4070 Ti Super GPU under Windows 10. To ensure reproducibility, the model configuration, camera matrices, scene parameters, analytical thresholds, and neural-network weights were stored separately from the resulting reports.

### 3.2. Synthetic Virtual Environment and Dataset Generation

**Figure 2:**
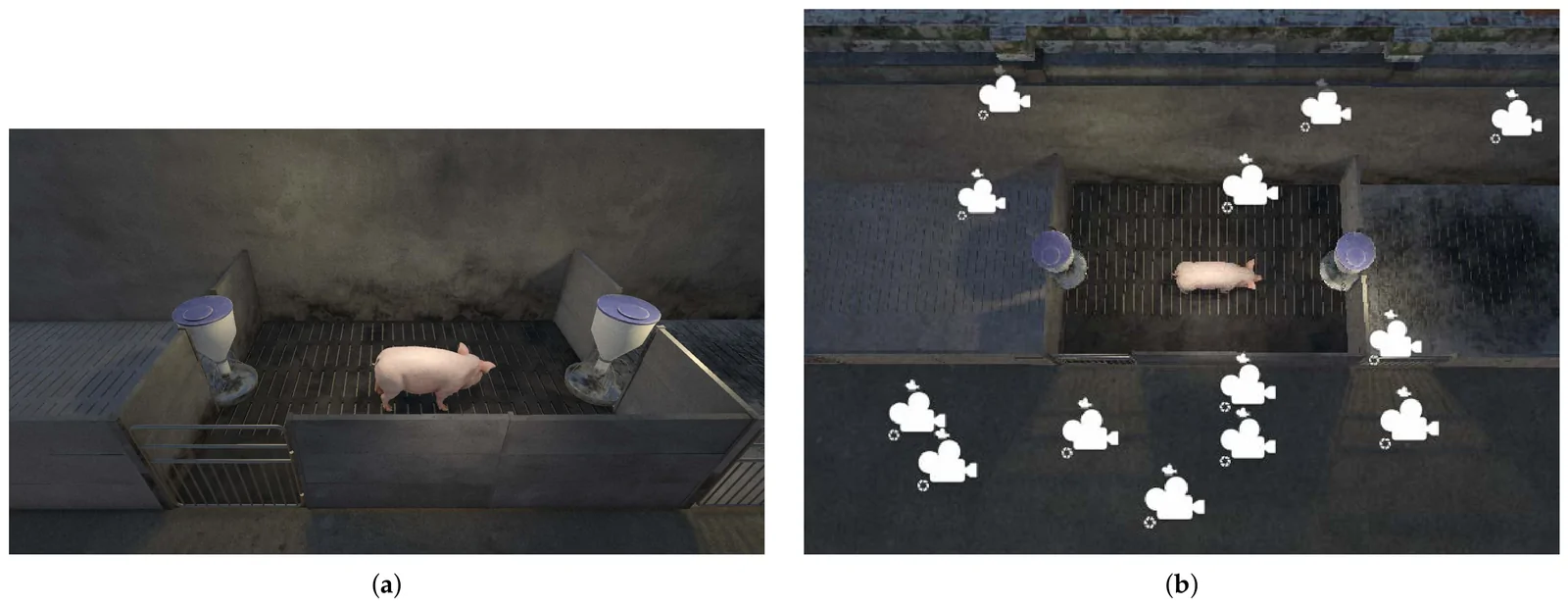

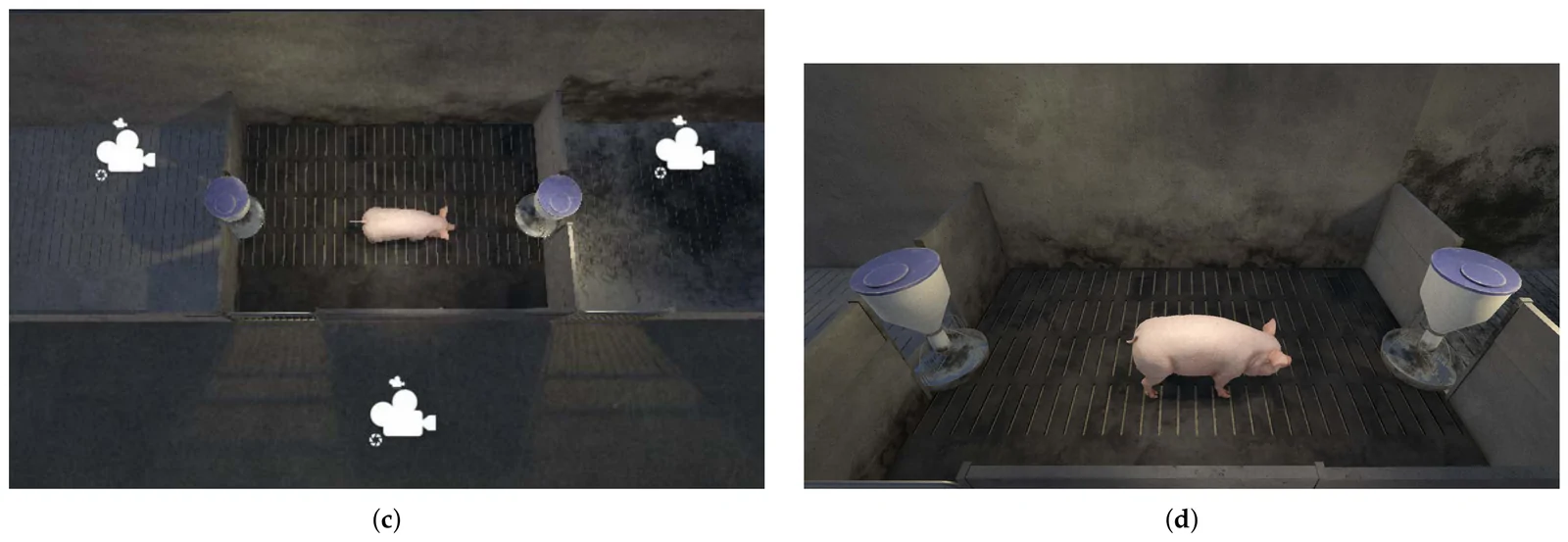
Synthetic virtual observation environment: (**a**) overall view of the individual-housing scene; (**b**) top view with twelve cameras used to generate static training images; (**c**) arrangement of the three held-out camera positions used for the synthetic video session; (**d**) oblique view of the pig model and the observation pen.

The synthetic virtual environment represented a three-dimensional individual-housing scene containing a parametric animated pig model, barriers, a slatted floor, feeding equipment, light sources, and a set of virtual cameras. During generation, the size, position, orientation, pose, and texture–material representation of the model were varied. Procedural generation was selected because synthetic data make it possible to obtain an image, camera parameters, and pose annotations from the same scene state without manual labeling. At the same time, a synthetic dataset may contain persistent artifacts of a particular engine and scene; therefore, contemporary reviews recommend randomizing the observation geometry, materials, illumination, and image-formation parameters [11,15]. The configuration of the virtual scene and the cameras used is shown in Figure 2.

The unit of generation was a synthetic three-dimensional state, denoted by the pose index. For each such state, the model position, rotation, and scale, the animation and normalized animation phase, the animal material, the environment variant, and the illumination configuration were selected once. The state was then rendered sequentially using twelve fixed cameras. Before each individual projection was saved, the field of view and post-processing parameters were selected independently. Consequently, images corresponding to the same pose index shared the same latent three-dimensional skeletal configuration but differed in perspective, self-occlusions, keypoint visibility, background, and optical effects. For the two-dimensional pose-estimation task, each projection remained a distinct image observation; however, all projections sharing the same pose index were assigned together to a single training, validation, or test subset.

Continuous parameters were sampled from uniform distributions using the UnityEngine.Random.Range function, whereas animations, environment variants, light colors, and film-grain types were selected with equal probability from the corresponding lists. The generated 150,000-image corpus and its annotations were retained. However, the numerical Unity random-number-generator seed/state used during its original procedural generation was not recorded. Therefore, the existing dataset can be reanalyzed directly, whereas exact procedural regeneration of the same corpus from the original random sequence is not claimed. This generator state is distinct from the grouped dataset-split seed (42) and the YOLO training seed (0).

Formally, the domain-randomization procedure was described as a stochastic transformation of virtual-scene parameters into an image and automatically generated annotations. For the *p*th three-dimensional state and the *c*th virtual camera, a synthetic sample was defined as

(1) $$D_{p,c}=\left(I_{p,c},b_{p,c},K_{p,c},\xi_{p},\eta_{p,c}\right),$$

where the image, bounding box, and set of keypoint annotations were supplemented with the parameters of the common three-dimensional state and those of the individual camera projection. The common-state vector included all parameters sampled once for a given pose index:

(2) $$\begin{array}{cc} \xi_{p}= & [r_{p}^{T},\psi_{p},s_{p},a_{p},\tau_{p},\mu_{p}^{T},\\ & e_{p},\mu_{p}^{(e)T},\ell_{p}^{T}{]}^{T}. \end{array}$$

The vector accounted for position, rotation, scale, animation and its phase, the animal-material parameters, a discrete environment variant, the environment-material parameters, and the aggregate characteristics of the light sources. The animal position within a rectangular region was defined by uniform distributions:

(3) $$\begin{array}{cc} x_{p} & ∼U\left(x_{0}-\frac{L_{x}}{2},\;x_{0}+\frac{L_{x}}{2}\right),\\ z_{p} & ∼U\left(z_{0}-\frac{L_{z}}{2},\;z_{0}+\frac{L_{z}}{2}\right),\\ y_{p} & ∼U\left(y_{0}-\frac{L_{y}}{2},\;y_{0}+\frac{L_{y}}{2}\right). \end{array}$$

The last distribution was used only when vertical randomization was enabled; in the analyzed configuration, the height remained fixed. Rotation about the vertical axis and isotropic scale were defined as

(4) $$\psi_{p}∼U\left(\psi_{\min},\psi_{\max}\right),\;s_{p}∼U\left(s_{\min},s_{\max}\right),$$

(5) $$q_{j,p}=r_{p}+R_{y}\left(\psi_{p}\right)s_{p}q_{j}^{0},\;R_{y}\left(\psi \right)=\left[\begin{array}{ccc} \cos\psi & 0 & \sin\psi \\ 0 & 1 & 0\\ -\sin\psi & 0 & \cos\psi \end{array}\right].$$

The animation state was sampled with equal probability from a finite set, and its normalized phase was sampled from a continuous uniform distribution:

(6) $$\begin{array}{cc} A & =\left\{A_{1},\ldots ,A_{N_{A}}\right\},\\ \mathrm{Pr}\left(a_{p}=A_{i}\right) & =\frac{1}{N_{A}},\\ \tau_{p} & ∼U(0,1),\\ Q_{p} & =F_{\mathrm{anim}}\left(a_{p},\tau_{p}\right). \end{array}$$

The animal-material modification parameters were defined by a vector containing hue, saturation, value, and smoothness transformations:

(7) $$\mu_{p}={\left[\Delta h_{p},k_{s,p},k_{v,p},\Delta \rho_{p}\right]}^{T},$$

(8) $$\begin{array}{cc} h_{p} & =\mathrm{mod}\left(h_{0}+\Delta h_{p},1\right),\\ s_{p}^{\left(m\right)} & =\mathrm{clip}\left(s_{0}k_{s,p},0,1\right),\\ v_{p}^{\left(m\right)} & =\mathrm{clip}\left(v_{0}k_{v,p},0,1\right),\\ \rho_{p} & =\mathrm{clip}\left(\rho_{0}+\Delta \rho_{p},0,1\right). \end{array}$$

Each component of the material vector was sampled independently from the corresponding range given in Table 1. The environment variant was also sampled from a discrete uniform distribution. When color randomization was enabled, an independent vector of the same type was applied to the materials of the selected environment set:

(9) $$\begin{array}{cc} E & =\left\{E_{1},\ldots ,E_{N_{E}}\right\},\\ \mathrm{Pr}\left(e_{p}=E_{i}\right) & =\frac{1}{N_{E}},\\ \mu_{p}^{\left(e\right)} & ={\left[\Delta h_{p}^{\left(e\right)},k_{s,p}^{\left(e\right)},k_{v,p}^{\left(e\right)},\Delta \rho_{p}^{\left(e\right)}\right]}^{T}. \end{array}$$

For each light source, the azimuth, radius, and height relative to the current animal center were sampled independently. The source position and direction toward a randomly displaced target point were defined as

(10) $$\alpha_{p,l}∼U\left(0,2\pi \right),\;r_{p,l}∼U\left(r_{\min},r_{\max}\right),\;h_{p,l}∼U\left(h_{\min},h_{\max}\right),$$

(11) $$\begin{array}{cc} \ell_{p,l} & =o_{p}+\left[\begin{array}{c} r_{p,l}\cos\alpha_{p,l}\\ h_{p,l}\\ r_{p,l}\sin\alpha_{p,l} \end{array}\right],\\ d_{p,l} & =\frac{o_{p}+\delta_{p,l}-\ell_{p,l}}{{∥o_{p}+\delta_{p,l}-\ell_{p,l}∥}_{2}}. \end{array}$$

The components of the target-point displacement were sampled independently from symmetric intervals. For spot and fill lights, the intensity and range were additionally varied; the cone angle was also varied for spot lights:

(12) $$\begin{array}{cc} I_{p,l}^{\left(\mathrm{spot}\right)} & ∼U\left(I_{\min}^{\left(\mathrm{spot}\right)},I_{\max}^{\left(\mathrm{spot}\right)}\right),\\ R_{p,l}^{\left(\mathrm{spot}\right)} & ∼U\left(R_{\min}^{\left(\mathrm{spot}\right)},R_{\max}^{\left(\mathrm{spot}\right)}\right),\\ \beta_{p,l} & ∼U\left(\beta_{\min},\beta_{\max}\right),\\ I_{p,l}^{\left(\mathrm{point}\right)} & ∼U\left(I_{\min}^{\left(\mathrm{point}\right)},I_{\max}^{\left(\mathrm{point}\right)}\right),\\ R_{p,l}^{\left(\mathrm{point}\right)} & ∼U\left(R_{\min}^{\left(\mathrm{point}\right)},R_{\max}^{\left(\mathrm{point}\right)}\right). \end{array}$$

The light color was sampled with equal probability from a predefined palette:

(13) $$\begin{array}{cc} C_{L} & =\left\{c_{1},\ldots ,c_{N_{L}}\right\},\\ \mathrm{Pr}\left(c_{p,l}=c_{i}\right) & =\frac{1}{N_{L}}. \end{array}$$

The parameters varied independently for each camera projection were combined into the vector

(14) $$\begin{array}{cc} \eta_{p,c} & ={\left[\phi_{p,c},\pi_{p,c}^{T}\right]}^{T},\\ \phi_{p,c} & ∼U\left(\phi_{\min},\phi_{\max}\right). \end{array}$$

where the field of view was supplemented with film-grain, chromatic-aberration, vignetting, color-correction, white-balance, and bloom parameters:

(15) $$\pi_{p,c}={\left[\begin{array}{c} g_{I},\;g_{R},\;g_{T},\;a_{\mathrm{chr}},\;v_{I},\;v_{S},\\ e,\;\kappa ,\;\sigma ,\;T,\;t,\;b \end{array}\right]}_{p,c}^{T}.$$

(16) $$\begin{array}{cc} g_{I,p,c} & ∼U\left(g_{I}^{\min},g_{I}^{\max}\right),\\ g_{R,p,c} & ∼U\left(g_{R}^{\min},g_{R}^{\max}\right),\\ a_{\mathrm{chr},p,c} & ∼U\left(a_{\mathrm{chr}}^{\min},a_{\mathrm{chr}}^{\max}\right),\\ v_{I,p,c} & ∼U\left(v_{I}^{\min},v_{I}^{\max}\right),\\ v_{S,p,c} & ∼U\left(v_{S}^{\min},v_{S}^{\max}\right),\\ e_{p,c} & ∼U\left(e_{\min},e_{\max}\right),\\ \kappa_{p,c} & ∼U\left(\kappa_{\min},\kappa_{\max}\right),\\ \sigma_{p,c} & ∼U\left(\sigma_{\min},\sigma_{\max}\right),\\ T_{p,c} & ∼U\left(T_{\min},T_{\max}\right),\\ t_{p,c} & ∼U\left(t_{\min},t_{\max}\right),\\ b_{p,c} & ∼U\left(b_{\min},b_{\max}\right). \end{array}$$

The film-grain type was sampled with equal probability from a finite set of built-in presets:

(17) $$\begin{array}{cc} G & =\left\{G_{1},\ldots ,G_{N_{G}}\right\},\\ \mathrm{Pr}\left(g_{T,p,c}=G_{i}\right) & =\frac{1}{N_{G}}. \end{array}$$

Thus, the common three-dimensional-state parameters were shared by all cameras, whereas the field of view and post-processing parameters were sampled independently for each projection. The joint parameter distribution was written as

(18) $$\begin{array}{cc} & p\left(\xi_{p},\eta_{p,1},\ldots ,\eta_{p,C}\right)\\ & \;=p\left(\xi_{p}\right)\prod_{c=1}^{C}p\left(\eta_{p,c}\right),\\ & \eta_{p,c_{1}}⊥\eta_{p,c_{2}}|\xi_{p}. \end{array}$$

The final image was produced by the rendering operator followed by post-processing:

(19) $$I_{p,c}=P\left(R\left(\xi_{p},C_{c},\phi_{p,c}\right),\pi_{p,c}\right).$$

Saving a synthetic observation was additionally constrained by requirements imposed on the width, height, and area of the bounding box and by the presence of at least one directly visible keypoint:

(20) $$\begin{array}{cc} V_{p,c}= & I\left[w_{p,c}^{\left(b\right)}\geq w_{\min}\right]I\left[h_{p,c}^{\left(b\right)}\geq h_{\min}\right]\\ & \times I\left[\frac{w_{p,c}^{\left(b\right)}h_{p,c}^{\left(b\right)}}{WH}\geq A_{\min}\right]I\left[N_{p,c}^{\left(\mathrm{visible}\right)}\geq 1\right]. \end{array}$$

A frame was included in the dataset only when the validity function was equal to one. Therefore, the actual distribution of stored observations was conditional on passing the validity check:

(21) $$\begin{array}{cc} p_{\mathrm{dataset}}\left(\omega \right) & =p\left(\omega |V(\omega )=1\right),\\ \omega & ={\left[\xi^{T},\eta^{T}\right]}^{T}. \end{array}$$

The limits of all continuous distributions and the numbers of discrete variants corresponded to the actual values stored in the serialized scene and are presented in Table 1.

**Figure 3:**
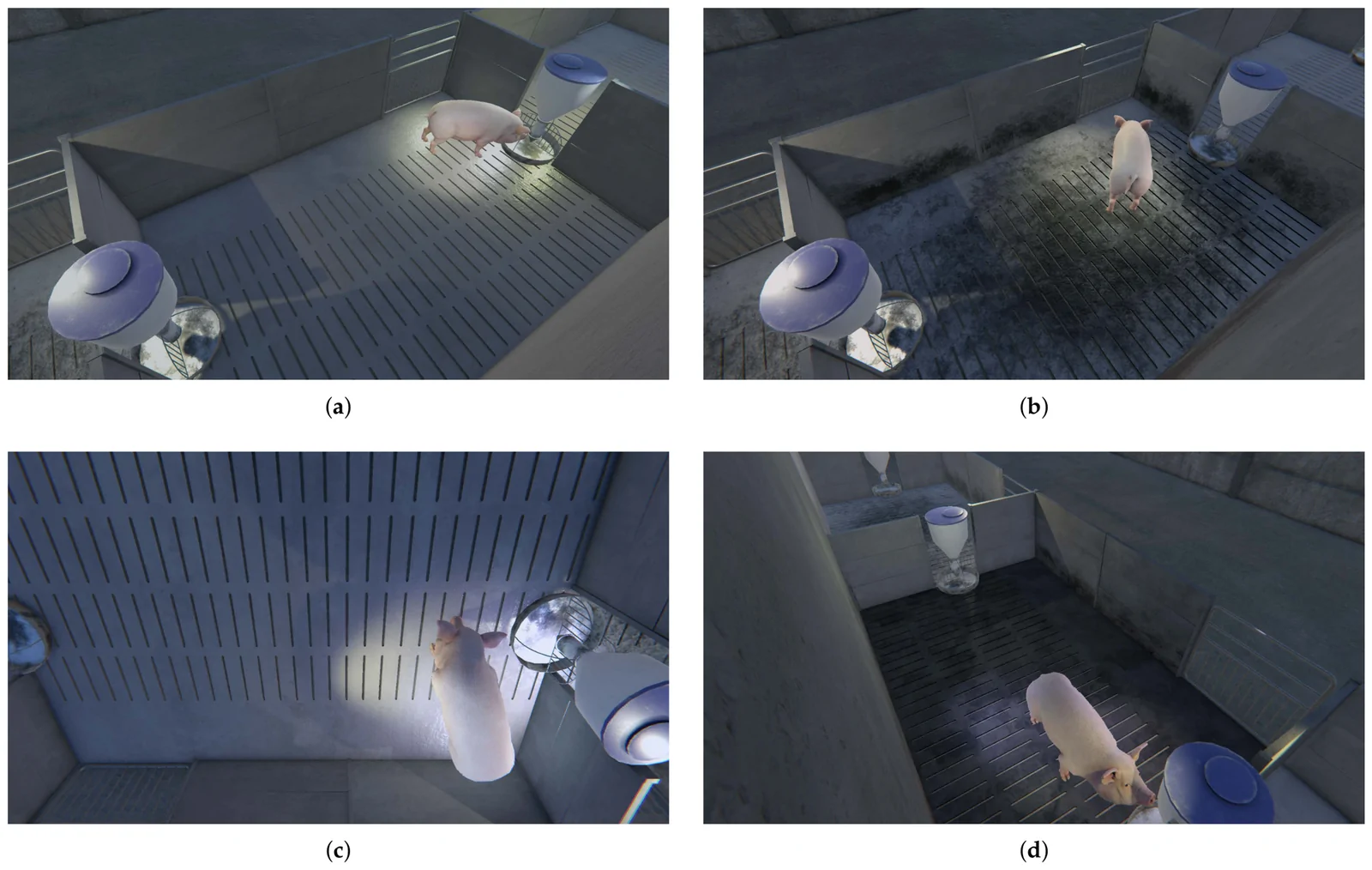
Examples of procedural variability in the synthetic dataset: (**a**–**d**) changes in model size and orientation, animation state, texture and material parameters, environment, illumination, field of view, and post-processing.

After an animation was selected, a random phase was assigned, root motion was disabled, and the animator was paused after synchronization. Before capture, two animator updates with a zero time step, two pose-stabilization frames, and one preliminary rendering pass were executed for each camera. As a result, each static training projection corresponded to a specific animation instant, whereas the global animal position was controlled separately. Examples of procedurally generated visual variability are presented in Figure 3.

**Figure 4:**
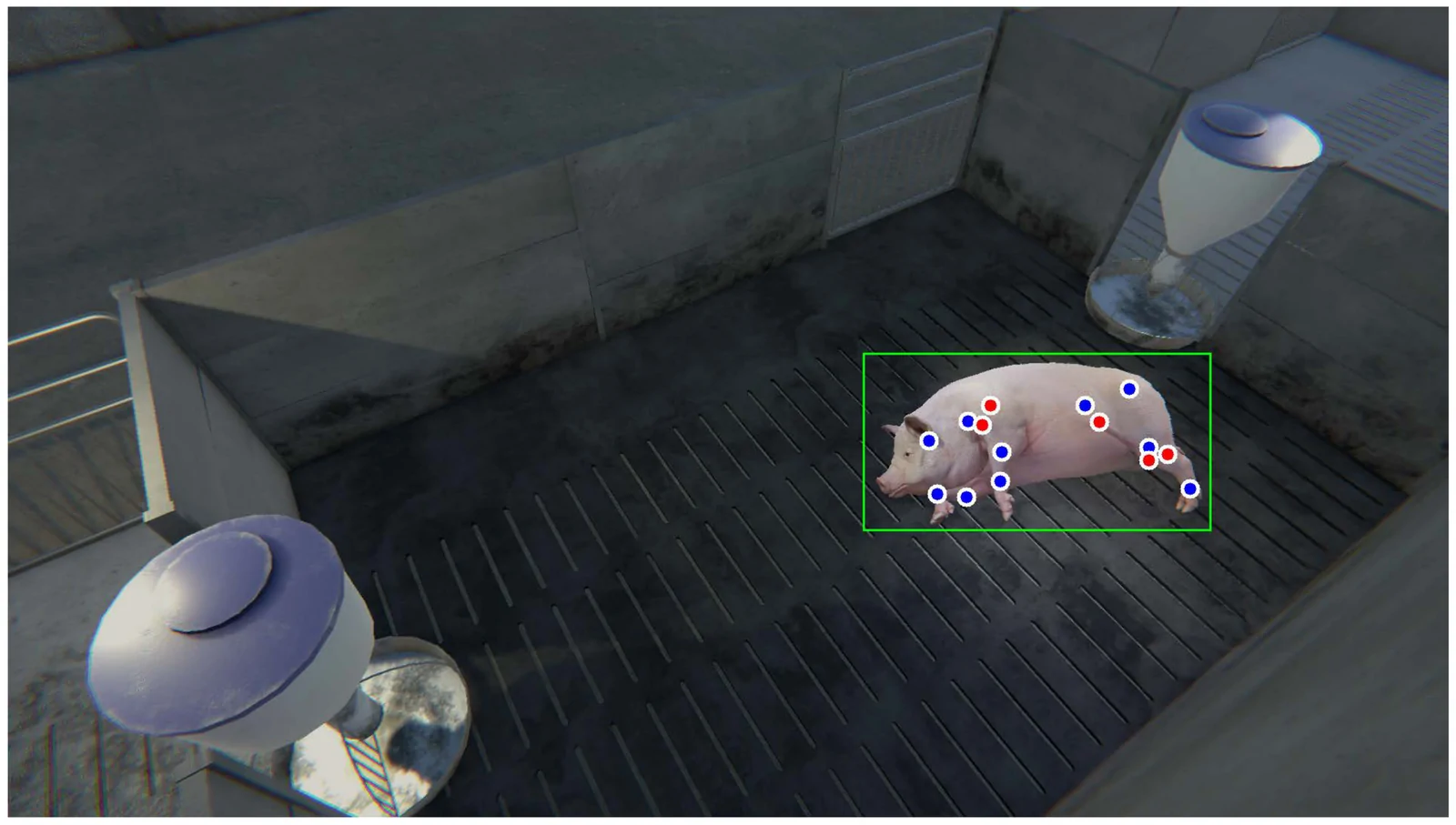
Automatic annotation system. The green rectangle denotes the automatically generated bounding box. Blue points denote keypoints directly visible to the camera, whereas red points denote keypoints occluded from the camera.

The output annotation of each image included one bounding box and fifteen semantically defined anatomical keypoints. This representation fully supported subsequent object detection, pose estimation, construction of a three-dimensional skeleton, and calculation of biomechanical indicators; pixel-level object segmentation was not required for the task. The keypoint order was fixed: 0, head; 1, neck; 2, spine/body center; 3–5, left shoulder, elbow, and front hoof; 6–8, right shoulder, elbow, and front hoof; 9–11, left hip, knee, and rear support point; and 12–14, right hip, knee, and rear support point. This skeleton preserved the trunk axis and four kinematic limb chains. A similar selection of functionally meaningful landmarks is used in studies of pig locomotion [8,9,10]. The annotation system and numbering of the anatomical landmarks are shown in Figure 4.

Visibility was determined using reverse projection and a ray-cast check between the camera and the keypoint. Code 0 was assigned to a point outside the frame or behind the camera, and its coordinates were set to zero in the YOLO annotation. Code 1 denoted a point located within the frame but occluded, for which the coordinates were retained. Code 2 corresponded to a directly visible point. The occlusion tolerance was 0.03 m. The ray-cast surface-hit tolerance was 0.20 m for external points and 0.65 m for internal points 0, 1, 2, 9, and 12. The bounding box was calculated from the projected vertices of the skinned mesh and expanded by 4 px. A projection was not saved if the bounding-box width or height was less than 8 px, the normalized area was less than 0.0005, or no keypoint was directly visible. If no projection was saved for a given state, the complete state was regenerated, with no more than 30 attempts.

The final dataset contained 150,000 PNG images representing 12,508 unique three-dimensional pose states. To prevent different camera projections of the same latent three-dimensional configuration from appearing in different subsets, the dataset was partitioned by pose index rather than by individual image. A grouped random split with seed 42 assigned 10,006 pose states (119,986 images) to the training subset, 1251 pose states (15,006 images) to the validation subset, and 1251 pose states (15,008 images) to an independent test subset. No pose index occurred in more than one subset. Each YOLO Pose annotation included the class identifier, normalized bounding-box parameters, and fifteen triples (x,y,visibility). The annotations file additionally contained the image, three-dimensional-state, and camera indices; filenames; field of view; post-processing parameters; animation and its phase; environment variant; model position, rotation, and scale; numbers of visible and occluded keypoints; bounding-box parameters; material color; and coordinates of all landmarks.

All synthetic training and evaluation data used the same parametric skeletal morphology. Variation was introduced through scale, pose, animation phase, materials, camera parameters, and scene conditions rather than through a second anatomical model.

### 3.3. Training and Evaluation of the Two-Dimensional Pose Model

The YOLO11m-pose architecture with pretrained initial weights was used for simultaneous animal detection and keypoint estimation. The input images were resized to 640 px, the batch size was 32, and four data-loading workers were used. The registered grouped run was performed for three epochs on CUDA device 0 using automatic optimizer selection, automatic mixed precision, seed=0, and deterministic mode. By epoch 2, pose precision and recall had reached 0.9976 and 0.9965, respectively, while pose ${\mathrm{mAP}}_{50–95}$ was 0.8494. The third epoch further increased pose ${\mathrm{mAP}}_{50–95}$ to 0.8999 and reduced the validation pose loss to 0.4655. The three-epoch schedule was retained as the predefined proof-of-concept training protocol; the independent test subset was not used to extend training or to tune hyperparameters. The optimizer=auto setting was retained to use the standard Ultralytics optimizer-selection procedure and to avoid dataset-specific manual tuning in this proof-of-concept experiment. Accordingly, the effective optimizer, initial learning rate, and momentum were determined internally by Ultralytics, whereas the final learning-rate multiplier and weight decay were set to 0.01 and 0.0005, respectively. The loss-component weights were 7.5 for the bounding box, 12.0 for pose, 1.0 for keypoint presence, 0.5 for class, and 1.5 for distribution focal loss. The training parameters are summarized in Table 2.

In addition to the variability already introduced during rendering, Ultralytics training transformations were applied: HSV modification (h=0.015,s=0.7,v=0.4), translation up to 0.1, scaling up to 0.5, mosaic augmentation with a probability of 1.0, RandAugment, and random erasing with a probability of 0.4. The shear and perspective parameters were zero; mixup, cutmix, and copy–paste were not used. All validation images were processed without test-time augmentation.

Model performance was evaluated using Ultralytics separately for bounding boxes and keypoints. Precision, recall, ${\mathrm{mAP}}_{50}$, and ${\mathrm{mAP}}_{50–95}$ were used; the latter averaged AP over matching thresholds from 0.50 to 0.95 with an increment of 0.05. For pose evaluation, correspondence was determined using the Ultralytics implementation of object keypoint similarity. The validation subset was used to monitor training performance, whereas the final model was additionally evaluated once on the independent pose-level test subset, which was not used for training or model selection. The Animal3D, MMVSL, and GANPose studies demonstrate the suitability of AP- and OKS-like indicators for anatomical-localization evaluation [9,10,12]; however, in the present study, these indicators characterize only the synthetic domain.

### 3.4. Multi-Camera Three-Dimensional Reconstruction and Quality Control

End-to-end evaluation was performed using three synchronized synthetic video sequences acquired from three cameras. They contained 27,000 synchronized temporal samples at 30 frames/s; the analyzed duration according to the timestamps was 899.967 s. The resolution was 1920×1080 px. Because the video streams were created in a consistent virtual scene, hardware synchronization was not required: the files began at the same virtual instant, frames were combined according to identical indices, and both the frame offset and timestamp offset in milliseconds were equal to zero for all cameras. Processing stopped when the shortest stream ended.

The cameras were represented using the OpenCV pinhole model. For camera *c*, transformation from the world coordinate system to the camera coordinate system, the projection matrix, and the relationship between a three-dimensional point and its two-dimensional image were defined as

(22) $$\begin{array}{cc} x_{i,c}^{\mathrm{cam}} & =R_{c}x_{i}^{\mathrm{world}}+t_{c},\\ P_{c} & =K_{c}\left[\begin{array}{cc} R_{c} & t_{c} \end{array}\right],\\ \lambda_{i,c}\left[\begin{array}{c} u_{i,c}\\ v_{i,c}\\ 1 \end{array}\right] & =P_{c}{\tilde{x}}_{i}. \end{array}$$

Here, $K_{c}$ is the intrinsic camera matrix, $R_{c}$ and $t_{c}$ are the extrinsic parameters, ${\tilde{x}}_{i}$ is the homogeneous coordinate vector of the keypoint, and $\lambda_{i,c}$ is a scale factor. The following common intrinsic matrix was used for all three cameras:

(23) $$K=\left[\begin{array}{ccc} 935.3074 & 0 & 960\\ 0 & 935.3074 & 540\\ 0 & 0 & 1 \end{array}\right].$$

Camera cam0 corresponded to the Unity object Main Camera (3), cam1 to Main Camera (12), and cam2 to Main Camera (13). These identifiers are persistent Unity object names and do not denote the twelve camera poses used for training-image generation. Before recording the end-to-end video sequence, the three camera objects were repositioned to locations that were not included among the twelve training camera positions. The extrinsic parameters were exported from Unity; the additional world transformation was the identity transformation, and the scale factor was 1. The complete matrices and vectors are presented in Table 3. The model weights and analytical thresholds were not adjusted for these recording positions.

At each time step, YOLO processed the cameras sequentially using a confidence threshold of 0.25. If the model returned several objects, the prediction with the highest bounding-box confidence was selected. For each anatomical keypoint, observations with a confidence of at least 0.25 from at least two cameras were used. Weighted linear triangulation was performed using the direct linear transformation method. For observation of keypoint *i* in camera *c*, the rows of the linear system were defined as

(24) $$\begin{array}{cc} a_{i,c}^{\left(u\right)} & =\sqrt{q_{i,c}}\left(u_{i,c}p_{c,3}^{T}-p_{c,1}^{T}\right),\\ a_{i,c}^{\left(v\right)} & =\sqrt{q_{i,c}}\left(v_{i,c}p_{c,3}^{T}-p_{c,2}^{T}\right). \end{array}$$

where $q_{i,c}$ is the keypoint confidence and $p_{c,j}^{T}$ is the *j*th row of the projection matrix. After the rows from all available cameras were combined, the homogeneous system

(25) $$\begin{array}{cc} A_{i}{\tilde{x}}_{i} & =0,\\ {\tilde{x}}_{i} & =v_{\min},\\ x_{i} & =\frac{1}{{\tilde{x}}_{i,4}}\left[\begin{array}{c} {\tilde{x}}_{i,1}\\ {\tilde{x}}_{i,2}\\ {\tilde{x}}_{i,3} \end{array}\right]. \end{array}$$

was solved, where $v_{\min}$ is the right singular vector of $A_{i}$ corresponding to its minimum singular value. A solution was rejected if the absolute value of the fourth homogeneous coordinate was less than $10^{-12}$ or if the point had non-positive depth in at least one camera used for the reconstruction [36,37].

When three observations were available, the solution using all cameras and all pairwise combinations were evaluated. The geometric consistency of a candidate was assessed using the Euclidean error between the original two-dimensional point and the reprojection of the reconstructed three-dimensional coordinate:

(26) $$\begin{array}{cc} e_{i,c} & ={∥\pi_{c}\left(x_{i}\right)-u_{i,c}∥}_{2},\\ {\bar{e}}_{i} & =\frac{1}{|C_{i}|}\sum_{c\in C_{i}}e_{i,c},\\ e_{i}^{\max} & =\max_{c\in C_{i}}e_{i,c}. \end{array}$$

For a three-camera solution, the maximum permissible mean reprojection error was 12 px; for a two-camera solution, it was 20 px. Among the admissible candidates, the solution with the minimum mean error was selected, followed by the minimum maximum error and a preference for a larger number of cameras. If no candidate satisfied the threshold, the keypoint was marked as high error and treated as technically invalid.

The three-dimensional trajectories were processed using two sequential filters. The first filter was designed to suppress isolated outliers and abrupt jumps, whereas the second applied exponential smoothing to improve temporal stability and suppress short-term trajectory jitter. These filtering stages were not intended to improve frame-aligned instantaneous three-dimensional positional accuracy.

(27) $$x_{i}^{\mathrm{clean}}\left(t\right)=\left\{\begin{array}{cc} x_{i}^{\mathrm{clean}}\left(t-1\right), & {∥x_{i}\left(t\right)-x_{i}^{\mathrm{clean}}\left(t-1\right)∥}_{2}>0.50,\\ 0.65\;x_{i}\left(t\right)+0.35\;x_{i}^{\mathrm{clean}}\left(t-1\right), & \mathrm{otherwise}, \end{array}\right.$$

(28) $$x_{i}^{\mathrm{smooth}}\left(t\right)=0.25\;x_{i}^{\mathrm{clean}}\left(t\right)+0.75\;x_{i}^{\mathrm{smooth}}\left(t-1\right).$$

The geometric center of the body, hereafter referred to as the body center, was defined as the unweighted mean of the available neck, spine, shoulder, and hip keypoints. An additional robust anchor combining the pelvic center and the spine keypoint was used for trajectory calculations:

(29) $$c_{g}\left(t\right)=\frac{1}{|B_{t}|}\sum_{i\in B_{t}}x_{i}^{\mathrm{smooth}}\left(t\right),\;B=\left\{1,2,3,6,9,12\right\},$$

(30) $$p_{\mathrm{pelvis}}\left(t\right)=\frac{x_{9}\left(t\right)+x_{12}\left(t\right)}{2},\;p_{a}\left(t\right)=0.7\;p_{\mathrm{pelvis}}\left(t\right)+0.3\;x_{2}\left(t\right).$$

The center $c_{g}$ was used as a geometric indicator of trunk position rather than as an anthropometrically weighted estimate of segmental mass distribution. Body orientation was determined from the direction from the spine toward the neck and the vertical world axis. The observation zone was represented by a 4.2×6.0 m rectangle with a floor height of 0.1015 m.

Technical reliability was evaluated separately from the simulated state. For each frame, the completeness of the three-dimensional skeleton, reprojection consistency, number of supporting cameras, and temporal stability of the keypoints were calculated. The component indicators and the final score were defined as

(31) $$Q_{\mathrm{valid}}=\min\left(\frac{N_{\mathrm{valid}}}{8},1\right),$$

(32) $$Q_{\mathrm{camera}}=\frac{1}{N_{\mathrm{valid}}}\sum_{i=1}^{N_{\mathrm{valid}}}\frac{\min(m_{i},3)}{3},$$

(33) $$Q_{\mathrm{temporal}}=1-\frac{1}{N_{v,\mathrm{frame}}}\sum_{i=1}^{N_{v,\mathrm{frame}}}\min\left(\frac{v_{i}}{3},1\right),$$

(34) $$Q=0.35Q_{\mathrm{valid}}+0.30Q_{\mathrm{reproj}}+0.15Q_{\mathrm{camera}}+0.20Q_{\mathrm{temporal}}.$$

Here, $m_{i}$ is the number of cameras supporting the *i*th keypoint, $v_{i}$ is its inter-frame velocity in m/s, and $N_{v,\mathrm{frame}}$ is the number of keypoints for which a valid inter-frame velocity estimate is available in the current frame; thus, the keypoint must have valid coordinates in both the current and preceding frames. The reprojection component decreased linearly between the soft and hard thresholds:

(35) $$Q_{\mathrm{reproj}}=\left\{\begin{array}{cc} 1, & \bar{e}\leq 6,\\ 1-\frac{\bar{e}-6}{15-6}, & 6<\bar{e}<15,\\ 0, & \bar{e}\geq 15. \end{array}\right.$$

A frame was classified as GOOD when Q≥0.80, ACCEPTABLE when 0.60≤Q<0.80, POOR when 0.35≤Q<0.60, and INVALID when Q<0.35 or fewer than eight valid keypoints were available.

An individual robust model of segment lengths was additionally constructed. For segment *j*, the baseline length and dispersion were estimated using the median and median absolute deviation:

(36) $${\tilde{l}}_{j}={\mathrm{med}}_{k}l_{j,k},\;{\mathrm{MAD}}_{j}={\mathrm{med}}_{k}\left|l_{j,k}-{\tilde{l}}_{j}\right|,$$

(37) $$\begin{array}{cc} \delta_{j} & =\frac{\left|l_{j}-{\tilde{l}}_{j}\right|}{\max\left({\tilde{l}}_{j},\varepsilon \right)},\\ z_{j}^{*} & =\frac{\left|l_{j}-{\tilde{l}}_{j}\right|}{\max\left({\mathrm{MAD}}_{j},0.005\right)}. \end{array}$$

The baseline model was updated only when the quality score was at least 0.70, the keypoint error did not exceed 10 px, and the point was supported by at least two cameras. The model was considered established after 5 s and at least 100 measurements. A warning was generated when the relative deviation exceeded 0.12 or the robust standardized error exceeded 4, whereas an anomaly was generated when the corresponding values exceeded 0.25 or 8. Joint-angle ranges, angular velocity, and a probable left–right interchange were also checked. These checks controlled authorization of subsequent calculations but did not directly modify the algorithmic state category.

The central quality gate authorized trajectory, walking, event, and baseline-model calculations at the GOOD level. At the ACCEPTABLE level, calculations were performed with a confidence multiplier of 0.85. At the POOR level, they were suspended, whereas at the INVALID level, they were blocked or rejected. This prevented a technical reconstruction error from being automatically transformed into an unfavorable state of the virtual model.

Direct spatial reconstruction accuracy was additionally evaluated on the separate 5-min scripted-ground-truth sequence. Each valid reconstructed landmark was compared directly with its exact Unity-world coordinate in the common world reference frame, without Procrustes alignment. Reconstruction coverage, mean per-joint position error (MPJPE), median Euclidean error, RMSE, and the 95th-percentile error were calculated. The same cached two-dimensional detections were also used to compare the fixed camera pairs, strict three-camera DLT, and the adaptive camera-selection strategy.

### 3.5. Temporal Analysis of Posture, Events, and Recovery Indicators

Posture features were extracted from the cleaned three-dimensional skeleton using a 0.60 s window. The mean shoulder and pelvic points, body length, median body height, and limb lowering were defined as

(38) $$s=\frac{x_{3}+x_{6}}{2},\;h=\frac{x_{9}+x_{12}}{2},\;L_{b}={∥s-h∥}_{2},$$

(39) $$H_{b}={\mathrm{med}}_{i\in B}y_{i},\;H_{l}={\mathrm{med}}_{i\in \{5,8,11,14\}}y_{i},\;D_{l}=H_{b}-H_{l},$$

(40) $${\hat{H}}_{b}=\frac{H_{b}}{L_{b}},\;{\hat{D}}_{l}=\frac{D_{l}}{L_{b}}.$$

Root velocity was calculated from displacement of the pelvic center in the horizontal plane, angular velocity from the change in the horizontal body axis, and limb-motion amplitude as the root-mean-square displacement in the body-fixed coordinate system:

(41) $$\begin{array}{cc} v_{r} & =\frac{{∥r\left(t_{1}\right)-r\left(t_{0}\right)∥}_{2}}{t_{1}-t_{0}},\\ \omega_{b} & =\frac{∠\left(a\left(t_{0}\right),a\left(t_{1}\right)\right)}{t_{1}-t_{0}}. \end{array}$$

(42) $$A_{l}=\sqrt{\frac{1}{\left|L\right|}\sum_{i\in L}{∥x_{i}^{\mathrm{local}}\left(t_{1}\right)-x_{i}^{\mathrm{local}}\left(t_{0}\right)∥}_{2}^{2}},\;L=\left\{5,8,11,14\right\}.$$

Continuous features were transformed into membership scores using a linear ramp function:

(43) $$\rho \left(x;a,b\right)=\left\{\begin{array}{cc} 0, & x\leq a,\\ \frac{x-a}{b-a}, & a<x<b,\\ 1, & x\geq b. \end{array}\right.$$

The lying, standing, walking, and turning scores were calculated as

(44) $$S_{\mathrm{lying}}=0.6\left[1-\rho \left({\hat{H}}_{b};0.58,0.82\right)\right]+0.4\left[1-\rho \left({\hat{D}}_{l};0.30,0.48\right)\right],$$

(45) $$S_{\mathrm{standing}}=0.6\rho \left({\hat{H}}_{b};0.58,0.82\right)+0.4\rho \left({\hat{D}}_{l};0.30,0.48\right),$$

(46) $$S_{\mathrm{walking}}=\min\left[S_{\mathrm{standing}},\sqrt{\rho \left(v_{r};0.048,0.080\right)\rho \left(A_{l};0.015,0.025\right)}\right].$$

(47) $$S_{\mathrm{turning}}=\min\left[S_{\mathrm{standing}},\rho \left(\omega_{b};15,25\right)\left(1-\rho \left(v_{r};0.06,0.09\right)\right)\right].$$

When the turning score exceeded 0.55, the walking score was additionally reduced in proportion to the residual score corresponding to the absence of turning. The minimum score for a candidate stable state was 0.55.

The finite-state machine distinguished unknown, lying, standing, walking, and the intermediate transition state. Entry into a regular state was confirmed for 0.35 s, entry into walking for 0.40 s, and exit from walking for 0.60 s. When valid features were unavailable, the current state was retained for no more than 0.50 s and then changed to unknown. A transition from lying to standing or walking was labeled rising, whereas the reverse transition was labeledlying down.

The baseline thresholds were fixed before the separate scripted-ground-truth evaluation and were not adjusted using its results. Spatial thresholds were expressed in physical units of the virtual scene, and temporal confirmation parameters were defined in seconds relative to the 30-Hz sampling rate. The quality-gate and event cutoffs were treated as engineering operating points for the present synthetic proof of concept rather than as biologically optimized clinical thresholds.

Critical events were determined from sequences of postures and three-dimensional coordinates rather than from a single image. The threshold values were defined as engineering criteria for the synthetic virtual environment and were applied without adaptation to the analyzed video session. Falls, convulsion-like movements, and prolonged-immobility episodes were considered. A prolonged-immobility episode was defined solely from a confirmed LYING state, limited displacement of the geometric body center, and accumulation of the required duration of valid immobility. No respiratory-micromovement feature was used by the implemented detector. All events represented algorithmic categories of the synthetic scenario and were not interpreted as clinical criteria. The operational definitions are summarized in Table 4.

For walking, the values 0.080 m/s and 0.025 m represent the saturation points of the corresponding membership ramps rather than the onset of the walking candidate. For fall detection, the 1.0-s interval specifies the maximum duration of the fall phase itself; the additional 0.35 s is required for confirmation of the subsequent LYING state. Therefore, $T_{\mathrm{tr}}\leq 1.35$ s denotes the complete candidate-to-confirmation interval, as formalized in Equation (48).

The formal conditions for fall registration combined the decrease in body-center height, maximum downward velocity, orientation change, transition duration, and subsequent confirmed lying:

(48) $$\begin{array}{cc} F(t)=I[ & \Delta h\geq 0.25\wedge v_{↓}^{\max}\geq 0.25\\ & \wedge \Delta \varphi \geq 15^{\circ }\wedge T_{\mathrm{tr}}\leq 1.35\\ & \wedge T_{\mathrm{lying}}\geq 0.35]. \end{array}$$

(49) $$\begin{array}{cc} C_{\mathrm{fall}}= & 0.45\min\left(\frac{\Delta h}{0.25},1\right)\\ & +0.40\min\left(\frac{v_{↓}^{\max}}{0.25},1\right)\\ & +0.15\min\left(\frac{\Delta \varphi }{15^{\circ }},1\right). \end{array}$$

A prolonged-immobility episode was defined by sustained recumbency with limited displacement of the geometric body center. Let $t_{0}$ denote the beginning of the current immobility candidate interval.

(50) $$D_{\mathrm{imm}}\left(t\right)=\max_{\tau \in [t_{0},t]}{∥c_{g}\left(\tau \right)-c_{g}\left(t_{0}\right)∥}_{2}.$$

(51) $$I\left(t\right)=I\left[S\left(t\right)=\mathrm{LYING}\wedge D_{\mathrm{imm}}\left(t\right)\leq 0.02\wedge T_{\mathrm{valid}}\left(t_{0},t\right)\geq 10\;s\right].$$

The detector initialized the candidate interval at the first valid LYING observation and confirmed the event only after 10 s of accumulated valid immobility. The stored event start time denotes the retrospectively assigned onset of the confirmed interval rather than the time at which the detector decision was emitted.

For spectral analysis of convulsion-like movement, the coordinates of eight limb joints and support points were transformed into the body-fixed coordinate system:

(52) $$z_{i}\left(t\right)=R_{b}^{T}\left(t\right)\left[x_{i}\left(t\right)-c_{g}\left(t\right)\right],$$

(53) $$V_{\mathrm{limb},\mathrm{RMS}}=\sqrt{\frac{1}{N_{v,\mathrm{limb}}}\sum_{\left(i,k\right)\in V_{\mathrm{limb}}}{∥\frac{z_{i}\left(t_{k+1}\right)-z_{i}\left(t_{k}\right)}{t_{k+1}-t_{k}}∥}_{2}^{2}}.$$

Here, $V_{\mathrm{limb}}$ is the set of valid limb-keypoint/inter-frame pairs included in the current analysis window, and $N_{v,\mathrm{limb}}=\left|V_{\mathrm{limb}}\right|$ is the total number of the corresponding valid limb-velocity vectors. Pairs containing a missing or invalid coordinate at either adjacent time point are excluded. If all eight limb keypoints are available throughout a window containing *K* temporal samples, then $N_{v,\mathrm{limb}}=8\left(K-1\right)$.

The multidimensional body-fixed signal was mean-centered, after which its first principal component was obtained by singular-value decomposition and used as the one-dimensional sequence $s_{k}$. Spectral analysis was performed using a real-valued discrete Fourier transform. Let $\Delta t_{\mathrm{med}}$ denote the median positive interval between valid successive samples.

(54) $$\begin{array}{cc} X_{m}=\sum_{k=0}^{N-1}s_{k}e^{-2\pi ikm/N}, & \\ f_{m} & =\frac{m}{N\Delta t_{\mathrm{med}}},\;\Delta f=\frac{1}{N\Delta t_{\mathrm{med}}},\\ m^{*} & =\underset{m:\;0.5\leq f_{m}\leq 8}{\arg\;\max}\left|X_{m}\right|,\\ f_{\mathrm{dom}} & =f_{m^{*}},\;N_{\mathrm{cycles}}=f_{\mathrm{dom}}\left(t_{\mathrm{last}}-t_{\mathrm{first}}\right). \end{array}$$

The implementation used a rolling buffer of up to 2.0 s. For a complete 2-s interval sampled at approximately 30 Hz, the nominal frequency resolution was therefore about 0.5 Hz. The frequency axis was constructed using the median positive sampling interval $\Delta t_{\mathrm{med}}$. Because the real FFT uses a single representative sampling interval, the spectral estimate assumes approximately uniform temporal spacing of valid samples within the analyzed buffer; the median interval accommodates small timestamp deviations from the nominal 30 Hz grid. Only frequency bins between 0.5 and 8 Hz were considered. No additional tapering window, zero-padding, or temporal resampling was applied. The cycle count was calculated using the actual time span between the first and last valid samples in the current buffer.

A candidate event was generated when the following conditions were satisfied:

(55) $$\begin{array}{cc} S(t)=I[ & S(t)=\mathrm{LYING}\\ & \wedge V_{\mathrm{limb},\mathrm{RMS}}\geq 0.08\\ & \wedge D_{\mathrm{root}}\leq 0.12\\ & \wedge \Delta \varphi \leq 30^{\circ }\\ & \wedge 0.5\leq f_{\mathrm{dom}}\leq 8\\ & \wedge N_{\mathrm{cycles}}\geq 2]. \end{array}$$

The conditions had to persist for at least 1.5 s. Recovery was confirmed for 0.75 s, after which a 3 s cooldown period was applied before repeated registration was allowed.

For quantitative representation of virtual recovery, each valid time point was assigned to one of four mutually exclusive categories. The hierarchy was applied in the following order: (1) a frame was assigned to Uncertain if the quality gate was POOR or INVALID or the posture was unknown; (2) within a technically valid interval, an active fall, convulsion-like movement, or prolonged immobility episode assigned the Bad category; (3) in the absence of an unfavorable event, stable standing, walking, or a confirmed rising transition assigned the Good category; and (4) calm recumbency not satisfying the prolonged-immobility or other critical-event criteria, together with other valid transitions, was assigned to Neutral. Technical events, including inconsistency of segment lengths, were not themselves classified as Bad and affected the result through the quality gate.

These four categories constitute a deterministic rule-based aggregation of posture, event, and measurement-quality outputs. Independent reference validation of the complete Good/Neutral/Bad hierarchy was not available. However, a restricted validation was performed for the independently referenceable posture-derived portion of the hierarchy on the second 5-min synthetic sequence. On both the manual-reference and algorithmic sides, standing and walking were mapped to Good, whereas lying was mapped to Neutral. Algorithmic frames with POOR or INVALID quality-gate status or unknown posture were recorded separately as Uncertain and treated as abstentions. These abstained frames were excluded from the Good/Neutral classification metrics and reported through prediction coverage. Bad was not assigned or scored in this restricted analysis because no independent adverse-event functional reference was available. Detector outputs and event thresholds were not used to construct reference Bad labels, thereby avoiding circular validation.

Let the durations of the mutually exclusive categories be $T_{\mathrm{good}}$, $T_{\mathrm{neutral}}$, $T_{\mathrm{bad}}$, and $T_{\mathrm{uncertain}}$. The total and functionally interpretable observation times were defined as

(56) $$\begin{array}{cc} T_{\mathrm{obs}} & =T_{\mathrm{good}}+T_{\mathrm{neutral}}+T_{\mathrm{bad}}+T_{\mathrm{uncertain}},\\ T_{\mathrm{valid}} & =T_{\mathrm{good}}+T_{\mathrm{neutral}}+T_{\mathrm{bad}}. \end{array}$$

The principal temporal indicators were calculated as

(57) $$\begin{array}{cc} P_{\mathrm{good}} & =100\frac{T_{\mathrm{good}}}{T_{\mathrm{valid}}},\\ P_{\mathrm{neutral}} & =100\frac{T_{\mathrm{neutral}}}{T_{\mathrm{valid}}},\\ P_{\mathrm{bad}} & =100\frac{T_{\mathrm{bad}}}{T_{\mathrm{valid}}},\\ P_{\mathrm{uncertain}} & =100\frac{T_{\mathrm{uncertain}}}{T_{\mathrm{obs}}}. \end{array}$$

When several critical events overlapped, the Bad duration was determined from the union of their temporal intervals without double counting. Indicators were calculated for the complete session and for sliding windows of 1, 5, and 15 min.

Additional descriptive indicators included total distance, distance per unit time, duration and number of walking episodes, mean and maximum velocity, number of posture transitions, number of events of each type, events per hour, and the maximum duration of a continuous unfavorable interval. For the sequence of accepted trajectory–anchor positions, distance and mean velocity were calculated as

(58) $$\begin{array}{cc} D & =\sum_{k\in A}\Delta r_{k},\\ \Delta r_{k} & ={∥p_{a}\left(t_{k}\right)-p_{a}\left(t_{k-1}\right)∥}_{2},\\ \bar{v} & =\frac{\sum_{k\in A}\Delta r_{k}}{\sum_{k\in A}\Delta t_{k}}. \end{array}$$

The set A included only intervals authorized by the quality gate and satisfying the spatial filters: displacements below 0.01 m and turns accompanied by a translation below 0.08 m did not increase the distance. The trajectory was aggregated into a heatmap with a cell size of 0.5 m; the cumulative occupancy duration was accumulated in each cell.

The statistical analysis was defined before interpretation of the results and was reproduced directly from the stored .csv and .json files. For the two-dimensional model, epoch-wise loss values, precision, recall, ${\mathrm{mAP}}_{50}$, and ${\mathrm{mAP}}_{50–95}$ were reported for the grouped validation subset, whereas the final precision, recall, ${\mathrm{mAP}}_{50}$, and ${\mathrm{mAP}}_{50–95}$ values were additionally calculated on the independent pose-level test subset. For the three-dimensional reconstruction, the number and completeness of temporal samples, mean ± standard deviation, median, interquartile range, and 95th percentile of the reprojection error, proportions of points supported by two and three cameras, and the distribution of quality-gate levels were calculated. For temporal states, the duration and proportion of the recording, number of transitions, kinematic indicators, and parameters of each event signal were reported.

Controlled component ablations were performed on the same cached two-dimensional detections and ground truth by removing confidence weighting, temporal filtering, the quality gate, and temporal state confirmation one component at a time. The pose-estimation model was not retrained for these comparisons.

To characterize temporal heterogeneity without treating individual frames as independent replicates, the 15-min recording was additionally divided into 15 non-overlapping 60-s blocks. For the minute-level proportions of Good, Neutral, Bad, and Uncertain, the mean ± standard deviation, median, interquartile range, and complete range were calculated. Empirical 95% temporal-stability intervals for the overall proportions were obtained using block bootstrap with replacement. In each of 10,000 iterations, 15 one-minute blocks were sampled randomly and the percentages were recalculated; a fixed seed of 20260715 was used. These intervals characterize variability among fragments of the analyzed synthetic session and are not confidence intervals for a population of animals.

Frames from the same sequence were not treated as independent observations. Therefore, Friedman or Wilcoxon tests, *p*-values, and between-group effect sizes were not calculated for this computational experiment.

A separate 5-min synthetic three-camera sequence was manually annotated at the times of changes among the stable states standing, walking, and lying. The annotations denoted as “lying after an attempt to stand” were retained as lying in the strict three-class frame-level comparison, while these intervals were additionally examined as transition attempts. Algorithmic labels were reconstructed from the state-transition records stored in the processing journal. The initial 0.367-s interval preceding confirmation of the first stable state was retained as unknown.

The reference timeline was produced by two observers working jointly in a single consensus annotation session. The sequence was reviewed frame by frame with reference to the corresponding synthetic animations. The onset of a new posture state was assigned to the first frame in which the visible motion was judged to indicate the beginning of that state, and the timestamp of that frame was recorded. Separate independent annotations were not produced; consequently, inter-rater agreement was not estimated. No prospective blinding protocol was documented, and the annotation is therefore not described as blinded.

For the additional reconstruction, sensitivity, and ablation analyses, a second 5-min synthetic evaluation sequence with exact geometric ground truth was generated in Unity 6000.4.5f1 in the *NewPig 1* scene using recording seed 12345. It comprised 9000 synchronized frames from three cameras at 30 fps and a resolution of 1920×1080 pixels. The retained evaluation record includes the recording and video manifests, complete three-camera calibration, per-frame Unity-world coordinates for all fifteen anatomical landmarks, and script-level animation and behavior intervals. The exact ground truth in this sequence refers primarily to the Unity-world geometry; the script-level intervals were retained as generation metadata and were not treated as independent semantic event labels for the fall or convulsion-like-movement detector classes. A separate manual posture reference was used for the temporal and functional-category evaluations.

Agreement was quantified using the frame-level confusion matrix, overall accuracy, class-wise precision, recall, and F1-score, macro- and duration-weighted F1-scores, and Cohen’s κ. Because the state machine intentionally applies temporal confirmation, a supplementary analysis was performed after excluding ±1.0 s neighborhoods of the reference transition times. For transition-level analysis, each reference transition was matched to the nearest subsequent algorithmic transition reaching the same target stable state within 5 s while preserving temporal order. This primary criterion evaluated detection of the transition boundary and the correct target state rather than requiring an exact source-to-target transition type. Matched transitions were counted as true positives, unmatched algorithmic transitions as false positives, and unmatched reference transitions as false negatives. The difference between matched algorithmic and reference timestamps was treated as the transition-confirmation delay. For this first manually annotated sequence, event detections were evaluated only for temporal compatibility with the posture reference. Independent event labels were not available for this sequence; therefore, event-level performance metrics were not calculated from it.

The second 5-min scripted synthetic sequence described above was additionally subjected to a limited manual temporal assessment. A separate visual annotation specified sixteen consecutive intervals of the stable states standing, walking, and lying, corresponding to fifteen reference transitions. Manual timestamps had whole-second resolution and were converted to seconds before matching. For this second-sequence analysis, reference and predicted transitions were matched one-to-one in temporal order only when both the source and target states agreed and the onset-time difference was within a symmetric ±5 s window. Matched transitions were counted as true positives, unmatched predicted transitions as false positives, and unmatched reference transitions as false negatives.

A limited prolonged-immobility reference was additionally derived from manually annotated lying intervals whose duration exceeded the implemented 10-s immobility criterion. Two such intervals were available. This reference therefore represents a consistency check against independently annotated posture intervals rather than an independently annotated clinical event. The script-level metadata indicated that no positive fall event occurred in this sequence. No independent semantic reference for convulsion-like movement was available.

Let the manual reference state at time point *t* be denoted by $s_{t}\in S$ and the algorithmic state by ${\hat{s}}_{t}\in S\cup \left\{\mathrm{unknown}\right\}$, where S={standing,walking,lying}. Strict frame-level accuracy was calculated over the complete sequence as

(59) $$A_{\mathrm{strict}}=100\frac{\sum_{t=1}^{N}I\left({\hat{s}}_{t}=s_{t}\right)}{N}.$$

The set of frames for which the state machine returned a stable functional state was defined as $V=\{t:{\hat{s}}_{t}\neq \mathrm{unknown}\}$. Prediction coverage and accuracy after exclusion of the initialization interval were calculated as

(60) $$C_{\mathrm{state}}=100\frac{\left|V\right|}{N},\;A_{\mathrm{known}}=100\frac{\sum_{t\in V}I\left({\hat{s}}_{t}=s_{t}\right)}{\left|V\right|}.$$

The elements of the frame-level confusion matrix were defined as

(61) $$M_{ij}=\sum_{t=1}^{N}I\left(s_{t}=i\wedge {\hat{s}}_{t}=j\right),i\in S,j\in S\cup \left\{\mathrm{unknown}\right\}.$$

Threshold robustness was evaluated on the second 5-min synthetic evaluation sequence using one-at-a-time group perturbations of −20%, −10%, +10%, and +20% relative to the baseline configuration, without retraining the pose model or adjusting the baseline parameters to the evaluation results. The analysis separately considered the state-membership ramps, quality-gate cutoffs, and the fall-detection threshold group. A total of 150 frames explicitly assigned to the transitional GoToRest and GoBackUp animations were excluded from the state-classification comparison, leaving 8850 classifiable frames. State accuracy, macro-F1, and prediction coverage were recalculated for each perturbation. Fall-threshold perturbations were interpreted as an output-stability check rather than as an estimate of event sensitivity, because the sequence contained no positive fall event.

## 4. Results

### 4.1. YOLO Training and Synthetic Validation

Training of YOLO11m-pose on the pose-index-grouped dataset was completed in 49,753.6 s, or 13.82 h. Over three epochs, the training pose loss decreased from 2.9373 to 1.0962, whereas the grouped-validation pose loss decreased from 1.3699 to 0.4655. Simultaneously, the validation keypoint ${\mathrm{mAP}}_{50–95}$ increased from 0.6647 to 0.8999. Similar dynamics were observed for animal localization: the validation bounding-box loss decreased from 0.8629 to 0.4346, whereas the bounding-box ${\mathrm{mAP}}_{50–95}$ increased from 0.7502 to 0.9085. The corresponding training and independent-test metrics are summarized in Table 5.

Most of the performance gain was obtained during the first two epochs: by epoch 2, pose precision and recall had reached 0.9976 and 0.9965, respectively, while pose ${\mathrm{mAP}}_{50–95}$ had increased to 0.8494. The third epoch further increased ${\mathrm{mAP}}_{50–95}$ to 0.8999 and reduced the validation pose loss to 0.4655. Thus, the grouped training run reproduced the rapid convergence observed previously, while the third epoch provided an additional improvement in keypoint localization accuracy.

**Figure 5:**
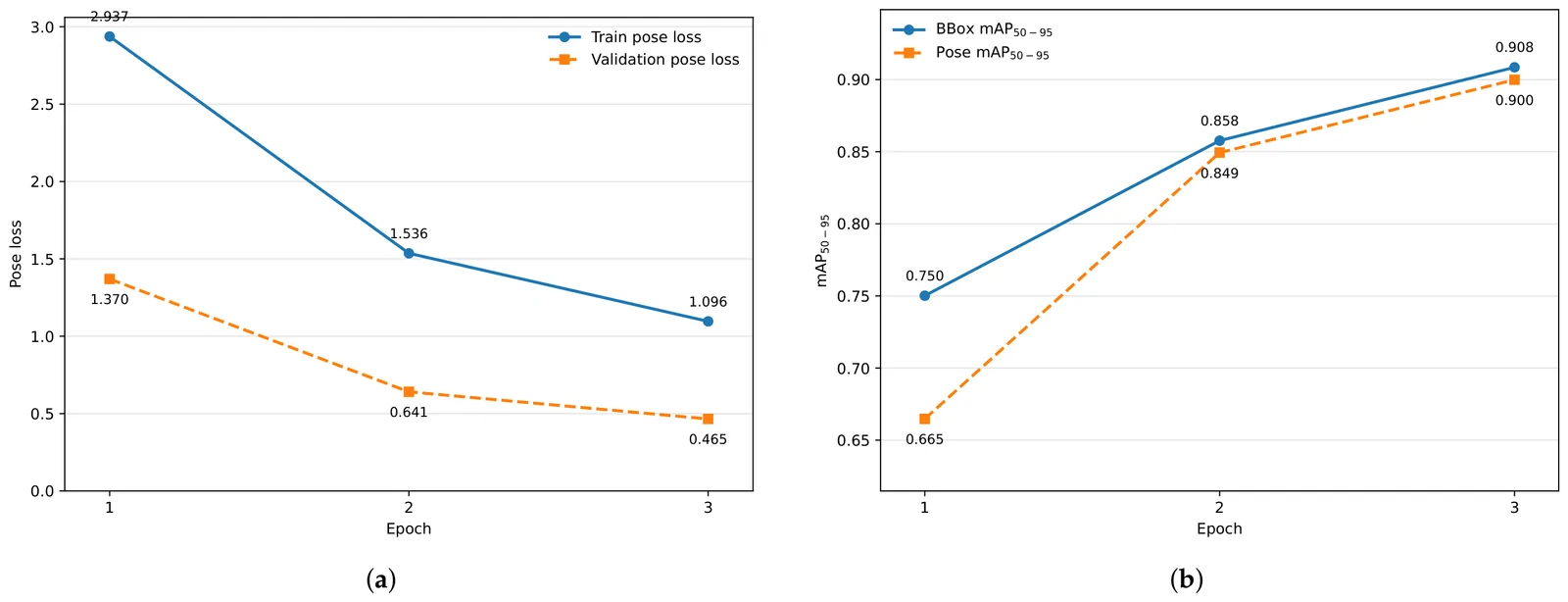
Training results of the two-dimensional model: (**a**) evolution of the training and validation pose losses; (**b**) evolution of the bounding-box and pose ${\mathrm{mAP}}_{50–95}$.

At the final validation epoch, pose precision was 0.9990, recall was 0.9983, ${\mathrm{mAP}}_{50}$ was 0.9950, and ${\mathrm{mAP}}_{50–95}$ was 0.8999. The best checkpoint was then evaluated on the independent pose-level test subset without additional training or parameter adjustment. Test pose precision was 0.9985, recall was 0.9986, ${\mathrm{mAP}}_{50}$ was 0.9950, and ${\mathrm{mAP}}_{50–95}$ was 0.8968. The test ${\mathrm{mAP}}_{50–95}$ was therefore only 0.0031 lower than the grouped-validation value. For bounding boxes, the corresponding independent-test precision, recall, ${\mathrm{mAP}}_{50}$, and ${\mathrm{mAP}}_{50–95}$ were 0.9996, 0.9997, 0.9950, and 0.9086, respectively. These results show that high pose-estimation performance was retained after eliminating cross-view pose leakage. These results refer to the pose-index-grouped validation and independent test subsets described above. The training dynamics are presented in Figure 5.

### 4.2. Three-Dimensional Reconstruction and the State Machine

A total of 27,000 synchronized temporal samples from three cameras were processed, corresponding to 900.0 s at 30 frames/s; the timestamp of the final frame was 899.967 s. A total of 405,000 records were stored, corresponding to 15 keypoints for each frame, with no missing rows. Of these, 168,823 points (41.685%) were reconstructed using three cameras and 236,177 points (58.315%) using the best camera pair. A valid status was assigned to 400,921 observations (98.993%), whereas 4079 observations (1.007%) were marked as errors.

**Figure 6:**
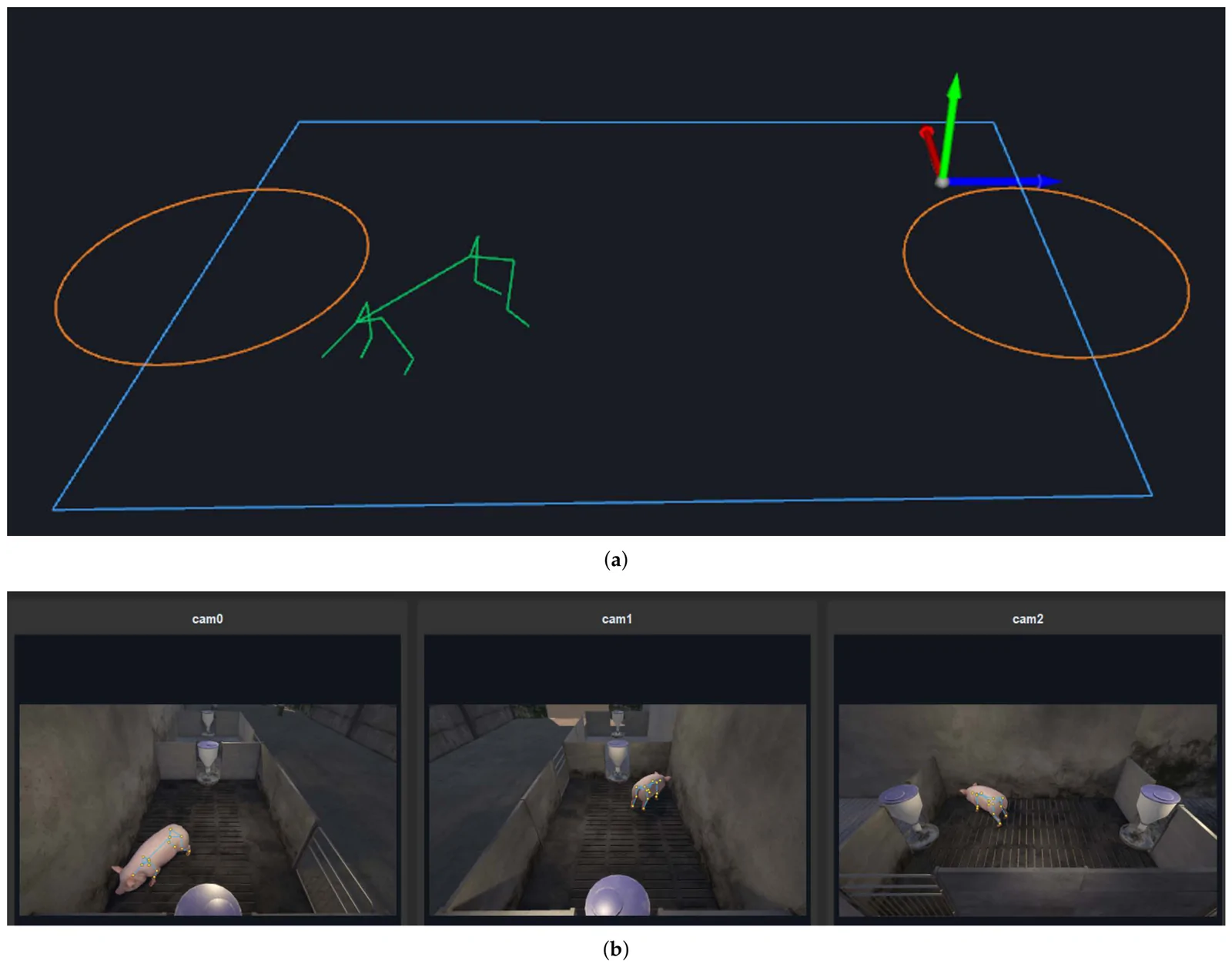
Multi-camera three-dimensional reconstruction: (**a**) reconstructed three-dimensional skeleton in the common world coordinate system;green segments denote the reconstructed skeleton, the light-blue outline indicates the pen boundary, orange circles indicate feeder regions, and the colored arrows show the coordinate axes; (**b**) synchronized frames from three virtual cameras with two-dimensional keypoint detections.

The mean reprojection error for an individual three-dimensional point was 3.689±3.671 px, the median was 2.420 px, the interquartile range was 1.489–4.287 px, and the 95th percentile was 11.382 px. The mean confidence of the two-dimensional keypoints was 0.9902±0.0327, with a median of 0.9983. The geometric tracking quality indicator had a mean value of 0.9367: 26,870 frames (99.519%) were classified as GOOD and 130 frames (0.481%) as ACCEPTABLE. The stricter central quality gate, which additionally accounted for the suitability of the skeleton for biomechanical calculations, produced a different temporal distribution: GOOD, 736.300 s (81.811%); ACCEPTABLE, 2.033 s (0.226%); POOR, 160.667 s (17.852%); and INVALID, 0.967 s (0.107%). The maximum duration of a single quality gap was only 0.533 s; however, 2083 short gaps cumulatively formed a substantial proportion of the uncertain time. An example of multi-camera reconstruction and the resulting three-dimensional skeleton is presented in Figure 6.

On the separate scripted-ground-truth sequence, 134,990 of 135,000 possible landmark instances were validly reconstructed, corresponding to 99.993% coverage. Direct comparison with the exact Unity-world coordinates yielded an MPJPE of 31.86 mm, a median error of 23.37 mm, an RMSE of 42.61 mm, and a 95th-percentile error of 88.74 mm. Mean frame-level error was 23.11 mm during standing, 27.91 mm during lying, and 37.84 mm during walking. Central body landmarks showed lower errors than distal limb landmarks.

Using identical cached two-dimensional detections, the fixed camera pairs cam0–cam1, cam0–cam2, and cam1–cam2 yielded MPJPE values of 33.78, 36.03, and 35.86 mm, respectively. Strict three-camera DLT yielded the lowest MPJPE of 29.46 mm with 99.336% coverage, whereas the adaptive strategy yielded an MPJPE of 31.86 mm with 99.993% coverage. Thus, strict three-camera reconstruction provided the highest spatial accuracy, while adaptive selection provided near-complete coverage and remained more accurate than the best fixed camera pair. The direct comparison of the reconstruction strategies is summarized in Table 6.

For the adaptive reconstruction used as the principal pipeline configuration, the median three-dimensional error was 23.37 mm, and the RMSE was 42.61 mm.

**Figure 7:**
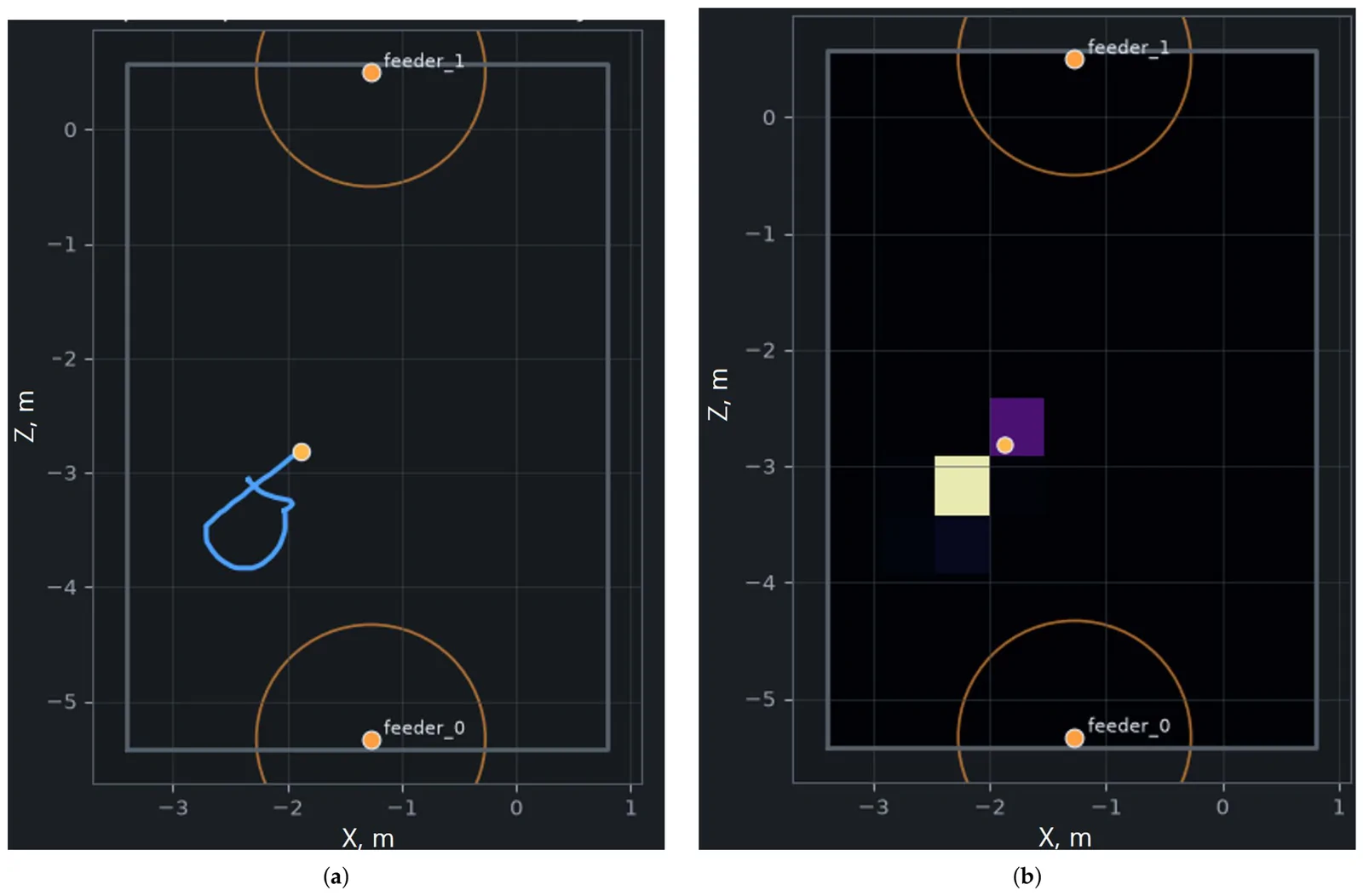
Spatiotemporal movement indicators: (**a**) trajectory of the geometric body center within the virtual pen; the blue line denotes the accepted trajectory, the gray rectangle indicates the pen boundary, and the orange markers and circles indicate feeder centers and nominal feeder regions; (**b**) occupancy-time heatmap, where lighter colors indicate longer occupancy.

The state machine assigned 470.800 s (52.311% of the recording) to standing, 212.767 s (23.641%) to walking, 216.033 s (24.004%) to lying, and 0.367 s (0.041%) to the unknown state. A total of 101 transitions between stable states were recorded. After application of the quality gate and trajectory filters, the duration of confirmed walking was 101.333 s across 49 episodes. The traveled distance was 11.584 m, the mean instantaneous velocity for valid walking samples was 0.378 m/s, and the maximum velocity was 1.006 m/s. The mean velocity was calculated from valid instantaneous values rather than as the ratio of the total distance to the entire duration of the walking state. The difference between the duration of the walking state and the time included in the distance calculation is expected because the latter additionally requires authorization by the quality gate and successful passage through the kinematic filters. The trajectory indicators and spatial distribution of activity are shown in Figure 7.

### 4.3. Fall, Prolonged-Immobility, and Convulsion-like Movement Signals

In the synthetic sequence, the algorithms generated ten signals related to the state of the virtual animal: one fall, six prolonged-immobility episodes, and three episodes of convulsion-like movement. In addition, 505 technical warnings concerning inconsistent segment lengths were registered. These warnings were not treated as unfavorable states and affected the result only through the quality gate.

The temporal values reported for prolonged-immobility episodes denote retrospective onset times. For example, the first valid LYING observation occurred at 0.333 s and initialized the candidate interval; the corresponding event was confirmed only after approximately 10 s of valid immobility, at about 10.333 s. The segment-length baseline described above is a separate quality-control procedure and is not a prerequisite for this event detector. The detected signals and their measured characteristics are summarized in Table 7.

For the three convulsion-like signals, the mean limb RMS velocity was 0.205 m/s, the mean dominant frequency was 2.131 Hz, and the root displacement ranged from 0.0006 to 0.0023 m. The maximum orientation change was $0.999^{\circ }$, which is consistent with the predefined rule for distinguishing local periodic limb movements from displacement and rotation of the entire body. The intervals generated by the individual detectors partially overlapped; therefore, the sum of their durations was not used directly. After interval union without double counting, the total duration of unfavorable events was 62.233 s. After technically uncertain fragments had been excluded, 61.333 s were assigned to the Bad category.

These values describe detector activations obtained in the 15-min scenario and demonstrate execution of the three event-detection modules; they should not by themselves be interpreted as event-level accuracy estimates. A limited manual temporal assessment of prolonged immobility was performed on the second 5-min synthetic sequence, as reported below. The fall and convulsion-like-movement detectors were not independently event-annotated in the available reference data.

### 4.4. Algorithmic Assessment of State Dynamics and Temporal Stability

Of the total 900.0 s recording, 162.0 s (18.000%) were assigned to Uncertain because of a POOR or INVALID quality gate status or the unknown state. The duration of the functionally interpretable observation was 738.0 s. Within this interval, Good accounted for 549.300 s (74.431%), Neutral for 127.367 s (17.258%), and Bad for 61.333 s (8.311%). Thus, the algorithmic profile of this synthetic scenario comprised 74.43% favorable and 8.31% unfavorable states within the valid observation time, with 18.00% technically uncertain time relative to the complete recording.

Analysis of 15 non-overlapping one-minute blocks demonstrated pronounced temporal heterogeneity within the scenario. For Good, the mean minute-level proportion was 75.64±26.67%, the median was 84.67%, and the range was 39.33–100.00%. For Bad, the corresponding values were 7.70±9.03%, 0%, and 0–20.59%. For Uncertain, they were 18.00±10.23%, 15.44%, and 7.39–38.78%, respectively. The wide intervals reflect the alternation of predefined movement fragments and periods of reduced observation quality rather than inter-individual biological variability. The complete temporal distribution of the algorithmic categories is summarized in Table 8.

### 4.5. Comparison with Ground-Truth State Annotations

The manually annotated test sequence contained 9000 synchronized temporal samples acquired at 30 Hz, corresponding to 300.0 s of observation. The exported dataset contained 135,000 three-dimensional keypoint records, with no missing spatial coordinates. Only 36 reconstructions (0.0267%) were marked as high-error cases. The median and mean reprojection errors were 2.381 and 3.112 px, respectively. Three-camera support was available for 48.45% of the points, whereas 51.55% were reconstructed from the best available camera pair (Table 9).

The strict frame-level agreement was 8208 of 9000 frames, corresponding to an accuracy of 91.20%. When the 0.367-s initialization interval assigned to unknown was excluded, accuracy was 91.31%, while algorithmic-state coverage was 99.88%. The macro-averaged F1-score was 90.10%, the duration-weighted F1-score was 91.50%, and Cohen’s κ was 0.860, indicating strong agreement between the manual and algorithmic state sequences. Class-wise state-recognition performance and state durations are summarized in Table 10.

The lying state was recognized most reliably, with a precision of 100.00% and an F1-score of 97.05%. The lower precision for walking (74.87%) resulted primarily from short transition movements. Relative to the ground truth, standing and lying were underestimated by 5.67 and 5.27 s, respectively, whereas walking was overestimated by 10.57 s.

**Figure 8:**
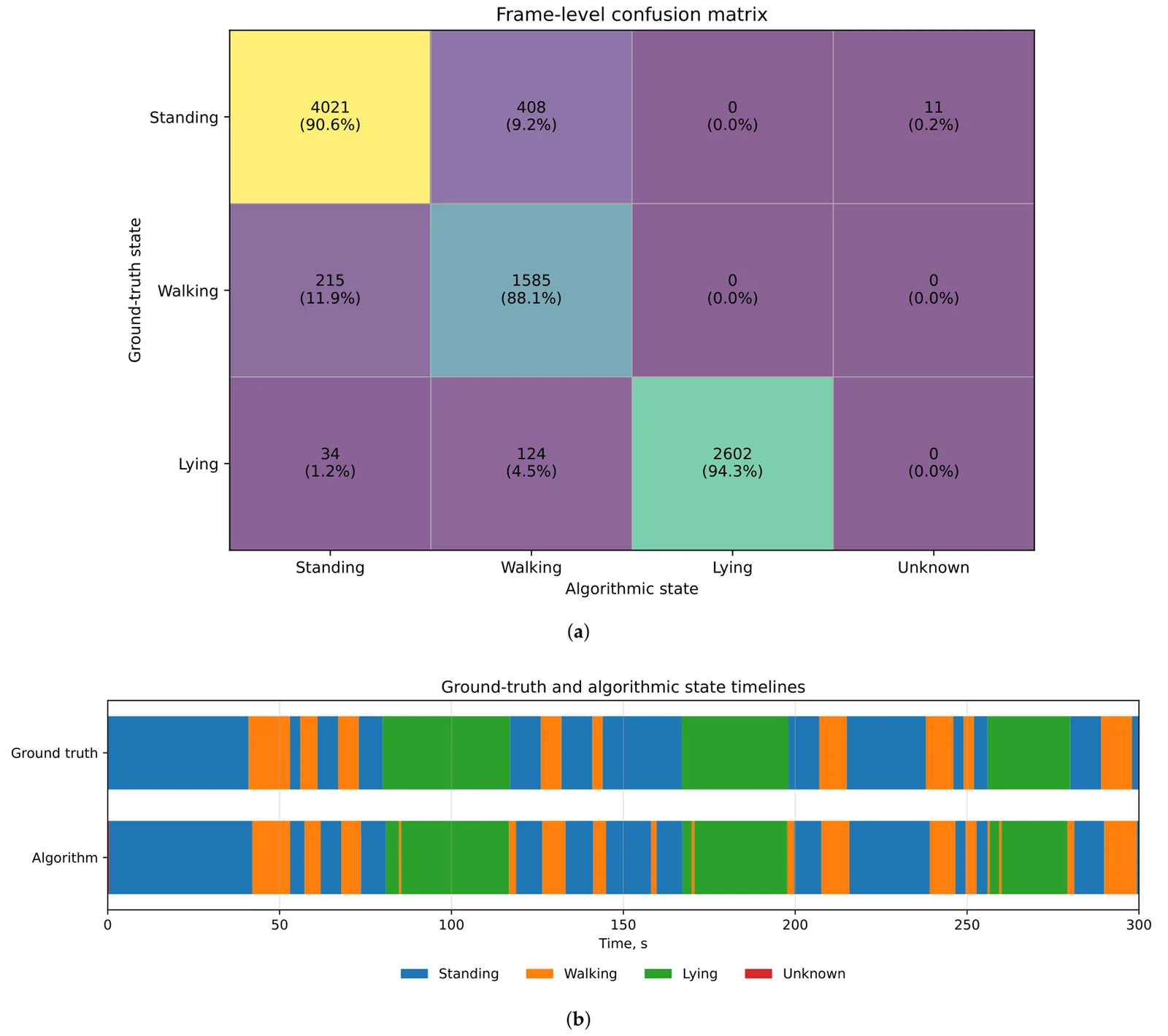
Comparison of the manual and algorithmic state annotations for the 5-min synthetic test sequence: (**a**) frame-level confusion matrix, with each cell showing the number of frames and the row-wise percentage; cell color intensity represents the number of frames; the exact count and row-wise percentage are reported within each cell. (**b**) categorical ground-truth and algorithmic state timelines. The unknown category represents the initial finite-state-machine confirmation interval rather than an animal state.

The most frequent disagreement was the assignment of ground-truth standing to walking (408 frames, 13.60 s), accounting for 51.5% of all mismatched frames. The reverse disagreement, walking classified as standing, comprised 215 frames (7.17 s; 27.1%). Ground-truth lying was classified as walking in 124 frames (4.13 s) and as standing in 34 frames (1.13 s). No standing or walking frame was falsely assigned to lying. The confusion matrix and complete categorical timelines are shown in Figure 8.

At the transition level, all 24 reference transitions were matched, whereas 12 additional algorithmic transitions remained unmatched. This corresponded to 24 true positives, 12 false positives, and no false negatives, giving a transition precision of 66.7%, recall of 100.0%, and F1-score of 80.0%. The false-transition rate was 2.4 min^−1^. The mean, median, and maximum confirmation delays for matched transitions were 0.868, 0.900, and 1.767 s, respectively. The additional transitions were concentrated around attempts to stand and short intermediate state assignments during posture changes, indicating temporal over-segmentation rather than missed reference transitions. In the supplementary frame-level analysis excluding ±1.0 s neighborhoods of the manually annotated transitions, state accuracy increased from 91.20% to 97.43%.

The three manually noted attempts to stand occurred at 84, 170, and 259 s. The algorithm assigned short walking intervals of 84.733–85.400, 169.867–170.667, and 259.367–260.033 s, respectively. These intervals were counted as lying-to-walking errors in the strict three-class comparison, although they coincided with manually recorded motor attempts and therefore reflected detected movement rather than random state substitution. Similar brief walking intervals preceded several successful transitions from lying to standing.

The journal additionally contained three prolonged-immobility signals, two convulsion-like-movement signals, and no fall signals. All five animal-event signals occurred within intervals manually annotated as lying and were therefore compatible with the posture ground truth. Because this first manually annotated sequence contained posture labels but no independent event labels, these activations were used only for posture-compatibility analysis and not for calculation of event-level performance metrics. Technical bone-length warnings (200 records) were analyzed separately and were not treated as changes in animal state.

On the second 5-min synthetic sequence, the manual reference contained 15 exact source-to-target transitions, whereas the algorithm generated 16 predicted transitions. Fourteen transitions were matched, corresponding to 14 true positives, two false positives, and one false negative. Transition precision was 87.5%, recall was 93.3%, and F1-score was 90.3%. The mean signed onset error was +0.860 s, onset MAE was 0.926 s, median absolute onset error was 0.850 s, and the maximum absolute onset error was 1.767 s. All sixteen manually annotated state intervals overlapped a predicted interval of the same state; the mean and median temporal IoU values were 0.895 and 0.913, respectively, with an onset MAE of 1.019 s and an offset MAE of 0.875 s.

Two manually annotated lying intervals, 17–40 s and 219–233 s, exceeded the implemented 10-s prolonged-immobility criterion and were used as limited reference intervals. Both were detected, yielding two true positives, no false positives, and no false negatives; thus, precision, recall, and F1-score were each 100% within this sequence, and the observed false-alarm rate was 0 h^−1^. The retrospectively assigned detector onset had an MAE of 1.317 s relative to the manual onset. Mean confirmation latency after the retrospectively assigned detector onset was 10.033 s, offset MAE was 0.750 s, and mean temporal IoU was 0.881. These results should be interpreted narrowly because only two positive prolonged-immobility intervals were available and the reference intervals were derived from manually annotated lying periods rather than independently annotated as clinical events.

The prolonged-immobility results in Table 11 represent a limited consistency assessment only. Manual timestamps had whole-second resolution, only two positive intervals were available, and the reference was derived from the duration of manually annotated lying intervals. The script-level metadata indicated that no positive fall event was present in this sequence, and the detector produced no fall activation; therefore, zero fall false alarms were observed during the 300-s recording (0 h^−1^). Because no positive fall event occurred, fall recall and F1-score were not estimable. No independent semantic reference was available for convulsion-like movement, and no class-level performance metrics are reported for that detector.

A restricted functional-category analysis was additionally performed on the same second 5-min synthetic sequence. Manual standing and walking frames were mapped to Good and manual lying frames to Neutral; the corresponding algorithmic posture-derived categories were obtained using the same mapping. Of 9000 reference frames, 8984 were covered (99.822%), whereas 16 frames (0.178%) were assigned to Uncertain and treated as abstentions. On the covered frames, selective Good/Neutral accuracy was 98.60% and binary macro-F1 was 96.67%. Good precision, recall, and F1-score were 98.80%, 99.61%, and 99.20%, respectively; the corresponding Neutral values were 97.03%, 91.42%, and 94.14%. Of the sixteen abstained frames, thirteen corresponded to unknown posture and three were rejected by the quality gate.

The class-wise precision, recall, and F1 values in Table 12 were calculated on the 8984 covered frames; the sixteen Uncertain frames were reported separately as abstentions. The resulting macro-F1 of 96.67% is therefore explicitly a binary Good/Neutral metric. Bad was not included in this analysis because no independent functional reference for the adverse-event branch was available. Accordingly, these results validate the internal consistency of the independently referenceable posture-derived portion of the functional hierarchy and do not constitute validation of the complete Good/Neutral/Bad classification.

On the second 5-min synthetic evaluation sequence, baseline state accuracy, macro-F1, and prediction coverage were 97.40%, 97.77%, and 99.83%, respectively, over 8850 classifiable frames. Perturbation of the state-membership ramps by ±10% and ±20% changed accuracy only within 96.97–97.57% and macro-F1 within 97.30–98.02%, while coverage remained 99.83%. Loosening the quality-gate cutoffs had negligible effect on coverage, whereas tightening them by 10% and 20% reduced coverage to 95.19% and 90.35%, respectively. Thus, the state-membership rules were comparatively insensitive to the tested perturbations, whereas prediction coverage was more sensitive to stricter quality-gate settings. The final Good/Neutral/Bad/Uncertain fractions remain deterministic descriptive outputs of the rule-based aggregation and are not interpreted as independently validated biological classes.

Event counts in Table 13 are reported only as output-stability observations. They are not interpreted as event-level sensitivity estimates, because the sensitivity sequence contained no positive scripted fall event and did not provide an independent positive reference for every event class.

Controlled ablations were additionally performed for confidence weighting, temporal filtering, the quality gate, and temporal state confirmation. The quantitative results are summarized in Table 14.

Confidence weighting was effectively neutral under the clean synthetic geometry, whereas temporal confirmation showed the clearest functional trade-off: its removal improved frame-level agreement but increased temporal fragmentation and produced a fall activation in a sequence containing no scripted falls. The increased frame-aligned error of the smoothed trajectory was caused primarily by temporal lag. Accordingly, smoothing is interpreted as a temporal-stability and short-term jitter-suppression step rather than as a method for improving instantaneous three-dimensional positional accuracy.

## 5. Discussion

### 5.1. Main Results and Scientific Contribution

The present study demonstrated the computational feasibility of a complete pipeline for the quantitative analysis of pig motor state, ranging from the procedural generation of training images and estimation of two-dimensional keypoints to multi-camera three-dimensional reconstruction, temporal posture classification, event detection, and calculation of the proportions of simulated recovery states. The principal result is not an individual YOLO accuracy value but the integration of these stages into a single system with a common timeline and explicit quality control. This approach transforms frame-level neural-network predictions into indicators that can be interpreted at the level of an observation session: the durations of standing, walking, and lying; distance; velocity; number of transitions; critical signals; and the proportions of Good, Neutral, Bad, and Uncertain states. Accordingly, the reported Good, Neutral, and Bad proportions are descriptive outputs of the predefined rule set and are not presented as diagnostic classes or probabilities of biological recovery.

The pose-index-grouped evaluation showed that the high synthetic-domain accuracy of YOLO11m-pose was retained after eliminating cross-view dependence between the training and evaluation subsets. On the independent test subset, keypoint ${\mathrm{mAP}}_{50–95}$ was 0.8968, compared with 0.8999 on the grouped validation subset. The small difference between these values indicates that the model performance was not dependent on different projections of the same latent three-dimensional pose being present across the data split. These results remain specific to the generated synthetic distribution and should not be interpreted as evidence of equivalent performance on real-animal images. Therefore, the obtained metrics characterize held-out synthetic observations within the generated distribution. Further evidence of the system’s operability was provided by integrating the model into a separate three-camera temporal sequence and constructing a complete three-dimensional skeleton for all 27,000 temporal samples.

The multi-camera reconstruction showed that 98.993% of the three-dimensional points satisfied the adopted geometric criteria, and the median reprojection error was 2.420 px. Only 41.685% of the points were supported by all three cameras, whereas the best camera pair was selected for 58.315%. This result demonstrates the practical role of redundant viewpoints: the third camera does not necessarily participate in every solution, but it increases the number of available camera pairs under occlusion, unfavorable geometry, or reduced confidence in an individual projection. This use of multi-camera redundancy is consistent with contemporary systems for three-dimensional animal pose estimation [36,37].

Direct comparison with the Unity-world ground truth refined this interpretation: strict three-camera DLT provided the lowest spatial error (MPJPE 29.46 mm), whereas the adaptive strategy provided near-complete coverage (99.993%) with an MPJPE of 31.86 mm and remained more accurate than any fixed two-camera pair. Thus, minimum reprojection error was used as an internal candidate-selection criterion and was not interpreted as evidence of minimum three-dimensional error.

Geometric acceptance and final uncertainty describe different units and stages of the processing pipeline. The 98.993% geometric-acceptance value is calculated for individual reconstructed keypoints, whereas the central quality gate operates at the frame level and additionally determines whether the reconstructed skeleton is sufficiently reliable for downstream biomechanical calculations. The original analyzed run did not retain per-component penalty traces. To examine the quality-gate mechanism separately, the available 900-s recording was diagnostically reprocessed with the current pipeline. Among 3087 frames classified as POOR in this diagnostic analysis, the dominant weighted penalty was camera support in 74.4%, temporal stability in 25.4%, reprojection consistency in 0.2%, and valid-point completeness in 0.0%. These diagnostic percentages explain the operation of the composite quality criterion and are not substituted for the quality-gate proportions reported for the original analyzed run.

The resulting algorithmic profile—74.43% Good, 17.26% Neutral, and 8.31% Bad within 738.0 s of valid observation time—represents a formalized description of a specific virtual scenario. The categories were defined by the author-developed classifier and do not indicate the probability of recovery. Their purpose is to transform heterogeneous postures and events into a mutually exclusive temporal representation suitable for comparing consecutive fragments. In particular, calm recumbency that did not satisfy the prolonged-immobility or other critical-event criteria was distinguished from prolonged immobility and convulsion-like movements; therefore, a lying posture alone did not automatically increase the proportion of the unfavorable state.

The first manually annotated 5-min test sequence demonstrated 91.20% strict frame-level state accuracy and a Cohen’s κ of 0.860. Accuracy increased to 97.43% outside ±1 s neighborhoods of the reference transitions, showing that most disagreements were concentrated around deliberately delayed finite-state-machine confirmations rather than within stable posture intervals. The principal residual error was an overestimation of walking during short transition movements and attempts to stand; introducing an explicit reference transition category would allow these motor episodes to be evaluated separately in future studies. The transition-level analysis also revealed temporal over-segmentation: transition recall was 100.0%, but precision was 66.7% because 12 additional short transitions were generated. Thus, the present confirmation logic favored detection of reference state changes at the cost of additional fragmentation around transition boundaries.

The second 5-min sequence provided an additional temporal check under a different state composition. Exact source-to-target transition matching yielded a precision of 87.5%, recall of 93.3%, and F1-score of 90.3%, with an onset MAE of 0.926 s. A limited prolonged-immobility assessment detected both of the two lying-derived reference intervals without false alarms in the 300-s recording. However, this result is based on only two positive intervals and does not constitute general validation of the event-detection subsystem. In particular, the fall and convulsion-like-movement detectors remain without independent event-level annotations in the present study.

### 5.2. Comparison with Existing Approaches

Most contemporary computer-vision studies in pig farming address two-dimensional detection, tracking, posture determination, or recognition of individual forms of behavior [4,5,6,7]. Such methods make it possible to estimate the presence and location of an animal and its discrete posture, but they do not always produce a continuous biomechanical representation linking limb movements, body-center motion, and temporal events. Pig pose-estimation models, including locomotion analysis, GANPose, and multimodal pose and action recognition, demonstrate the value of anatomical landmarks for more detailed behavioral analysis [8,9,10]. The present study extends this direction by transitioning from two-dimensional pose to a calibrated three-dimensional skeleton and subsequently calculating indicators over the entire recording.

A direct numerical comparison of the obtained mAP with the results reported in [8,9,10] would be inappropriate. The studies differ in their keypoint sets, numbers of animals, occlusion complexity, dataset splitting strategies, and types of source images. In the present work, training and end-to-end evaluation were conducted in a virtual environment; therefore, the high YOLO metrics should be interpreted as evidence of the learnability of the selected annotation system and the effectiveness of the generated synthetic variability rather than as universal superiority over other architectures.

The use of synthetic data is particularly important for events that are rare, hazardous, or poorly represented in conventional video datasets. Reviews of synthetic datasets emphasize the advantages of accurate automatic annotation and controlled scene variation [11,15]. In the proposed pipeline, the synthetic virtual environment made it possible to define the model size and position, animation, textures and materials, illumination, camera parameters, and post-processing, while automatically obtaining landmark coordinates and visibility information. This reduces annotation costs, enables the dataset to be scaled to 150,000 frames, and allows a controlled distribution of rare scenario events to be generated.

Critical-event detection in the proposed system is based on interpretable temporal features rather than on opaque assignment of a single class to an individual frame. A fall is associated with a decrease in the geometric body center, downward velocity, an orientation change, and subsequent lying; a prolonged immobility episode is associated with a confirmed lying state, limited displacement of the geometric body center, and persistence of these conditions over the specified interval; and convulsion-like movement is associated with local limb velocities and the spectral structure of the signal. This design makes it possible to store the measured features together with the signal and analyze the reason for each activation. Overlap between individual events demonstrated the need to merge unfavorable intervals rather than sum detector durations. Similarly, 505 segment-length consistency violations were classified as technical events rather than animal-state events. This prevents reconstruction errors from being conflated with the state of the virtual object.

Unlike studies in which the final output is the accuracy of posture or behavior-class recognition, the proposed system generates the temporal structure of simulated recovery. Such aggregation is consistent with the general transition from single-event detection to long-term behavioral monitoring [4,5,14]. The scientific novelty lies in combining synthetically trained pose estimation, multi-camera three-dimensional geometry, controlled rejection of unreliable decisions, and event-based logic into a unified author-developed quantitative indicator that is interpretable within the predefined virtual scenario.

### 5.3. Applicability of the Developed Software Pipeline

The proposed pipeline is intended for the automatic analysis of synthetic multi-camera video sessions generated in a synthetic virtual environment. For each recording, the system generates a temporal state diagram, a trajectory and occupancy heatmap of the geometric body center, traveled distance, number of walking episodes, velocity, intervals corresponding to prolonged immobility episodes, and fragments surrounding critical events. All indicators are calculated in a common coordinate system and stored together with an assessment of technical quality.

Although the present synthetic scenario was formulated in terms of recovery, the pipeline operates on generic kinematic and temporal features and can be adapted to disease, farrowing, welfare assessment, and other prolonged monitoring tasks by redefining the state labels and event thresholds.

The most informative representation is not a single label but the dynamics of category proportions in consecutive temporal windows. An increase in Good, a decrease in Bad, a change in distance, and a reduction in uncertain time make it possible to quantitatively compare different synthetic scenarios or consecutive fragments of the same video session. Repeated falls, prolonged immobility episodes, convulsion-like movements, and reduced motor activity are represented as separate events. The Uncertain category is reported alongside the principal percentages because a change in the result accompanied by a simultaneous deterioration in reconstruction quality should not be interpreted as a change in the state of the virtual object.

The developed system is a software and analytical classifier rather than a clinical diagnostic system. Fall and convulsion-like-movement outputs are treated as detector activations within the synthetic scenarios and are not presented as independently validated event classifications because independent positive event-level reference annotations were not available for these classes. Prolonged-immobility detection was subjected only to the limited consistency assessment described above. The separation between state categories and technical uncertainty ensures explainability and makes it possible to trace which measured features led to each decision.

The synthetic generator can be used for targeted dataset expansion and balancing of scenario events. Within the synthetic virtual environment, combinations of viewpoint, occlusion, illumination, model size and texture, posture, and post-processing parameters can be varied independently, after which new visual situations can be added to the training dataset. This approach allows falls, convulsion-like movements, prolonged immobility episodes, and abnormal postures to be generated in a controlled manner without restrictions imposed by the frequency of rare classes. More generally, the development and application of synthetic datasets to problems of analysis, information processing, and control across a wide range of subject domains is a highly promising direction, as also demonstrated by related research [38].

The current implementation is primarily suitable for offline analysis. Processing a 15-min three-camera recording required approximately 68.6 min, corresponding to 6.56 synchronized frame sets per second and an approximately 4.57-fold slowdown relative to real time. This performance may be acceptable for monitoring after completion of a recording, but acceleration is required for immediate notification. Promising directions include batched YOLO inference for the three cameras, parallel frame preparation, transfer of triangulation matrix operations and temporal-feature calculations to the GPU, and asynchronous saving of event images. These changes can be implemented without reducing the metric-calculation frequency or the accuracy of the SVD- and FFT-based detectors.

### 5.4. Limitations of the Scope of the Study

The principal limitation of the study is its fully synthetic nature. All images, video sequences, camera positions, keypoints, and scenario events were generated in the synthetic virtual environment. Therefore, the obtained results characterize the operability of the software architecture, the internal consistency of the computations, and the author-developed classification of virtual states. Transfer to physical images and clinical interpretation are outside the scope of the present study. Accordingly, agreement with the manual and scripted reference data used in the synthetic evaluations quantifies internal synthetic-domain validity only and should not be interpreted as diagnostic accuracy or as evidence of equivalent performance on real-animal recordings.

The end-to-end video evaluation used camera positions that were not included among the twelve training-image viewpoints. This provides a test of camera-position variation within the same synthetic scene and parametric animal model; it does not establish generalization to different environments or animal morphologies. Procedural randomization was intended to reduce dependence on persistent scene-specific appearance cues; however, the present dataset was generated using one parametric animal model and one pen geometry with a limited set of material variants. Robustness to substantially different animal morphologies, scene geometries, independently held-out appearance domains, or independently held-out motion repertoires was not evaluated and is therefore not claimed.

A dedicated independent development set for optimization of the analytical thresholds was not retained. Accordingly, the reported thresholds are treated as predefined engineering operating points rather than empirically optimized values. Their local robustness was instead assessed on the separate scripted-ground-truth sequence using the predefined one-at-a-time sensitivity analysis, without test-time threshold adjustment.

The dataset was partitioned at the level of the underlying three-dimensional pose index so that all camera projections of a given pose state remained within a single training, validation, or test subset. The independent-test metrics were close to those obtained on the grouped validation subset. Nevertheless, all subsets originate from the same procedural generator and parametric animal model, and the reported performance therefore remains specific to the predefined synthetic distribution.

The end-to-end result was obtained from one 15-min three-camera video session. Two separate 5-min synthetic evaluation sequences were additionally used: the first for manual state-sequence comparison and the second for direct three-dimensional ground-truth evaluation, sensitivity and ablation analyses, the limited manual temporal assessment, and restricted Good/Neutral functional-category validation. None of these sequences was intended to support statistical generalization to a biological population.

Functional-category validation was restricted to the independently referenceable posture-derived Good/Neutral branch. On the second 300-s synthetic sequence, manual standing and walking were mapped to Good and manual lying to Neutral, while algorithmic Uncertain outputs were treated as abstentions. Bad had no independent functional reference and was therefore neither assigned nor scored; consequently, no Bad precision, recall, F1-score, or three-class macro-F1 is reported. The resulting Good/Neutral metrics quantify the internal consistency of the independently referenceable portion of the predefined hierarchy and should not be interpreted as validation of the complete functional classification, biological recovery state, or diagnostic performance.

Event-level validation also remains limited. On the second 5-min synthetic sequence, only two prolonged-immobility reference intervals were available, and these intervals were derived from manually annotated lying periods exceeding the implemented 10-s duration criterion rather than from an independent clinical event annotation. Manual timestamps had whole-second resolution. Independent fall and convulsion-like-movement annotations and a second independent annotation pass were not available. Consequently, the present results do not establish general sensitivity or specificity of all critical-event detectors.

The synthetic video streams were exactly synchronized, the camera parameters were defined by the virtual environment, and the distortion coefficients were zero. These conditions form part of the computational-experiment design and enable evaluation of the multi-camera geometry under a known acquisition configuration. Therefore, the reprojection errors and quality-gate indicators should be interpreted within the parameters of the virtual cameras used.

The threshold values of the critical-event detectors and state machine were defined as engineering criteria for the present synthetic proof of concept. In the implemented pipeline, a prolonged-immobility episode is identified from a confirmed LYING state, limited displacement of the geometric body center, and persistence of these conditions over the specified interval. This event should not by itself be interpreted as confirmation of death. At the same time, prolonged immobility represents one of the motor signs that can be incorporated into a future multi-criteria detector of severe and potentially lethal conditions. Such an extension would require additional physiological indicators, for example validated respiratory or other vital-function measures, together with evaluation against reference observations obtained from real animals. Thus, the present detector is considered an intermediate algorithmic component toward automated recognition of critical conditions rather than a validated mortality detector.

The one-minute blocks and bootstrap intervals characterize the temporal heterogeneity of a single sequence. They do not represent intervals of inter-individual variability and were used only to assess the stability of category proportions with respect to the composition of temporal fragments within the analyzed synthetic session.

The difference between geometric tracking quality and 18.0% uncertain time reflects the different levels at which these measures operate: geometric acceptance is evaluated for individual reconstructed points, whereas the final quality gate is a frame-level criterion governing suitability for downstream analysis. Component-level penalty traces were not retained for the original analyzed run; the separate diagnostic reprocessing therefore serves only to clarify the mechanism of the composite quality gate.

Thus, the presented work constitutes a complete synthetic proof of concept. It encompasses procedural dataset generation, training of the object and keypoint detector, multi-camera reconstruction, calculation of trajectory indicators, critical-event detection, and author-developed classification of recovery dynamics. The conclusions are limited to the predefined virtual domain; evaluation on physical images, transfer between synthetic and real domains, and clinical validation were not objectives of the present study.

## 6. Conclusions

This study proposed and implemented an end-to-end approach for the quantitative assessment of simulated pig motor state that integrates a synthetic virtual environment, procedural data generation, YOLO Pose training, multi-camera three-dimensional reconstruction, a state machine, and temporal detection of critical events. The generated synthetic dataset comprised 150,000 images annotated with fifteen keypoints and included variations in model size and texture, posture, viewpoint, illumination, materials, and post-processing parameters. After pose-index-level separation of the training, validation, and test subsets, the retrained YOLO11m-pose model achieved a keypoint ${\mathrm{mAP}}_{50–95}$ of 0.8968, a precision of 0.9985, and a recall of 0.9986 on the independent synthetic test subset. The corresponding grouped-validation ${\mathrm{mAP}}_{50–95}$ was 0.8999, confirming that high pose-estimation performance was retained after eliminating cross-view pose leakage within the predefined virtual domain.

The operability of the complete pipeline was evaluated using a 15-min synthetic sequence acquired from three synchronized cameras. A total of 27,000 temporal samples were processed, producing 405,000 three-dimensional keypoint records. Of the reconstructions, 98.993% satisfied the admissible geometric criteria, and the median reprojection error was 2.420 px. The state machine assigned 52.31% of the recording to standing, 23.64% to walking, and 24.00% to lying, and 101 transitions between states were recorded. The confirmed trajectory included 49 walking episodes with a total distance of 11.584 m. The event-detection algorithms generated one fall signal, six prolonged-immobility episode signals, and three convulsion-like-movement signals. On the second 5-min synthetic sequence, both of the two lying-derived prolonged-immobility reference intervals were detected without false alarms, with an onset MAE of 1.317 s and a mean temporal IoU of 0.881. Because only two such intervals were available and fall and convulsion-like-movement events were not independently annotated, these results are treated as a limited temporal consistency assessment rather than as validation of all event-detector classes. Technical violations of skeletal consistency were processed separately and were not interpreted as changes in the state of the virtual object. In the first manually annotated 5-min synthetic test sequence, the state machine achieved a strict frame-level accuracy of 91.20%, a macro-F1 score of 90.10%, and a Cohen’s κ of 0.860; accuracy outside ±1 s neighborhoods of state transitions was 97.43%.

After application of the quality gate, the functionally interpretable time was 738.0 s of the 900.0 s recording. Within this interval, the proportion of Good was 74.43%, Neutral was 17.26%, and Bad was 8.31%; a further 18.00% of the complete recording was assigned to Uncertain. On the second 5-min synthetic sequence, the posture-derived Good/Neutral classification achieved a selective accuracy of 98.60%, a binary macro-F1 of 96.67%, and prediction coverage of 99.822%. Good and Neutral F1-scores were 99.20% and 94.14%, respectively. Because no independent functional reference was available for the Bad branch, these results demonstrate only the internal consistency of the posture-derived Good/Neutral classification and do not validate the complete functional hierarchy. Uncertain remains a technical abstention rather than an animal state.

The principal methodological result is that simulated recovery dynamics are represented by an explicit rule-based temporal distribution rather than by a single frame-level label. Calm recumbency is distinguished from prolonged immobility and other critical-event conditions, overlapping events are accounted for without double counting, and a loss of observation quality is not transformed into a false unfavorable state.

The practical significance of the proposed approach lies in the development of a complete software pipeline for the quantitative analysis of synthetic recovery scenarios. The system automatically generates the temporal dynamics of the author-defined categories, the trajectory and occupancy heatmap of the geometric body center, distance, velocity, and fragments associated with critical events. The synthetic generator enables controlled reproduction of rare scenarios, including falls, convulsion-like movements, prolonged immobility episodes, and abnormal postures, while providing accurate automatic annotations for training the neural-network model. The prolonged-immobility detector may subsequently serve as one component of a multi-criteria system for recognizing severe and potentially lethal conditions, provided that additional physiological indicators and real-animal reference data are incorporated.

The conclusions are limited by the fully synthetic study design, one 15-min end-to-end sequence, two separate 5-min synthetic evaluation sequences, predefined event thresholds, and the author-developed classification scheme. Within these boundaries, the study represents a complete computational proof of concept demonstrating the internal consistency and technical feasibility of the system. The work does not claim transferability to physical images, clinical validity, or diagnostic interpretation of the calculated categories. Nevertheless, models trained on synthetic data have substantial potential for subsequent application under real-world conditions.

## Figures and Tables

**Table 1:** Domain-randomization parameters and actual values used in the serialized synthetic scene.

Parameter Group	Distribution and Actual Scene Values
Animal position	*x* from −2.91 to 0.13 m; *z* from −4.415 to −0.285 m; *y* fixed at 0.128 m
Orientation and size	Rotation about the vertical axis, 0–$360^{\circ }$; isotropic scale, 0.90–1.10
Animation	18 states selected with equal probability; normalized phase, 0–1
Animal appearance	Hue shift *H*, −0.125 to 0.125; saturation multiplier *S*, 0.80–1.20; value multiplier *V*, 0.80–1.30; smoothness change, −0.25 to 0.25
Environment	Three material sets selected with equal probability; additional independent HSV and smoothness randomization over the same ranges
Spot illumination	Seven independent sources; radius, 1.56–2.88 m; height, 1.00–2.61 m; intensity, 2–10; range, 4–12 m; cone angle, 25–$80^{\circ }$
Fill illumination	One effectively unique point light; intensity, 0.10–3.50; range, 2–8 m; shadows disabled
Light color	Eight discrete RGB colors ranging from neutral white to warm, turquoise, violet, and cool-blue tones
Camera	Twelve fixed and substantially different positions; perspective projection; random field of view of 55–$65^{\circ }$ for each image
Rendering	1920×1080 px; near clipping plane, 0.3 m; far clipping plane, 1000 m
Post-processing	Film-grain intensity, 0–0.25, and response, 0.60–0.90; chromatic aberration, 0–0.12; vignette intensity, 0–0.18, and smoothness, 0.20–0.45
Color correction	Post-exposure, −0.35 to 0.35; contrast, −10 to 15; saturation, −12 to 12; temperature, −10 to 10; tint, −5 to 5; bloom, 0–0.15

**Table 2:** Parameters used to train the two-dimensional pose-estimation model.

Training Parameter	Value
Architecture	YOLO11m-pose
Input size	640 px
Number of keypoints	15×3
Training/validation/test images	119,986/15,006/15,008
Epochs	3
Batch size	32
Optimizer	Automatic selection by Ultralytics
Initial learning rate and momentum	Determined automatically
Final learning-rate multiplier/ weight decay	0.01/0.0005
Seed/deterministic mode	0/enabled
Dataset split seed	42
Automatic mixed precision	Enabled
Device	NVIDIA GeForce RTX 4070 Ti Super, CUDA 12 (NVIDIA, Santa Clara, CA, USA)

**Table 3:** Extrinsic parameters of the three virtual cameras at the held-out positions used for end-to-end video evaluation.

Camera	Rotation Matrix *R*, by Rows	Translation Vector *t*, m
cam0	(1,0,0); (0,−0.7876452,−0.6161290); (0,−0.6161290,0.7876452)	${\left(1.270302,-0.8241684,7.275754\right)}^{T}$
cam1	(−1,0,0); (0,−0.7876452,0.6161290); (0,−0.6161290,−0.7876455)	${\left(-1.270302,2.075344,3.569085\right)}^{T}$
cam2	(−0.00000012,0,1); (0.6161290,−0.7876453,0); (−0.7876454,−0.6161290,−0.00000012)	${\left(2.454508,1.657482,4.103270\right)}^{T}$

**Table 4:** Operational definitions of temporal events and movement indicators.

Event or Indicator	Operational Definition
Fall	Decrease in the geometric body center by at least 0.25 m within a window of up to 1.0 s; maximum downward velocity of at least 0.25 m/s; orientation change of at least $15^{\circ }$; subsequent lying for at least 0.35 s; 3 s cooldown
Prolonged immobility episode	Confirmed LYING state; displacement of the geometric body center not exceeding 0.02 m; at least 10 s of accumulated valid immobility
Convulsion-like movement	Recumbent posture; 2 s window; limb RMS velocity of at least 0.08 m/s; dominant frequency of 0.5–8 Hz; at least two cycles and 1.5 s confirmation
Convulsion-like-event constraint	Displacement of the geometric body center not exceeding 0.12 m and orientation change not exceeding $30^{\circ }$; recovery confirmation for 0.75 s; 3 s cooldown
Walking	Standing geometry combined with root-speed and limb-motion membership ramps of 0.048–0.080 m/s and 0.015–0.025 m, respectively; full membership is reached at 0.080 m/s and 0.025 m, followed by temporal confirmation
Distance	Sum of displacements of the geometric body center greater than 0.01 m only over intervals authorized by the quality gate

**Table 5:** Training, grouped-validation, and independent-test metrics of YOLO11m-pose. The independent-test values correspond to the final selected checkpoint.

Metric	Epoch 1	Epoch 2	Epoch 3	Independent Test
Train pose loss	2.9373	1.5360	1.0962	—
Validation pose loss	1.3699	0.6407	0.4655	—
BBox precision	0.9863	0.9994	0.9997	0.9996
BBox recall	0.9883	0.9990	0.9998	0.9997
BBox ${\mathrm{mAP}}_{50}$	0.9946	0.9950	0.9950	0.9950
BBox ${\mathrm{mAP}}_{50–95}$	0.7502	0.8577	0.9085	0.9086
Pose precision	0.9729	0.9976	0.9990	0.9985
Pose recall	0.9646	0.9965	0.9983	0.9986
Pose ${\mathrm{mAP}}_{50}$	0.9719	0.9949	0.9950	0.9950
Pose ${\mathrm{mAP}}_{50–95}$	0.6647	0.8494	0.8999	0.8968

**Table 6:** Direct three-dimensional reconstruction accuracy for fixed, strict three-camera, and adaptive reconstruction strategies using identical two-dimensional detections and exact Unity-world ground truth.

Reconstruction Strategy	MPJPE, mm	P95 Error, mm	Coverage, %
cam0 + cam1	33.78	101.11	99.997
cam0 + cam2	36.03	99.86	99.990
cam1 + cam2	35.86	97.92	99.958
Strict three-camera DLT	29.46	85.60	99.336
Adaptive reconstruction	31.86	88.74	99.993

**Table 7:** Signals detected in the synthetic video sequence and their measured characteristics.

Signal Type	Number	Temporal Parameters	Measured Features
Fall	1	191.167–192.033 s; transition duration, 0.867 s	Decrease in the geometric body center, 0.434 m; maximum downward velocity, 0.579 m/s; orientation change, $17.02^{\circ }$
Prolonged immobility episode	6	Retrospective onset times: 0.333, 194.767, 353.867, 484.233, 551.267, and 746.967 s; accumulated valid immobility, 10.000–10.833 s	Confirmed LYING state; displacement of the geometric body center not exceeding 0.02 m; event decision after satisfaction of the 10-s valid-immobility criterion
Convulsion-like movement	3	203.733–205.233, 554.800–556.300, and 749.133–750.633 s; duration, 1.500 s each	Limb RMS velocity, 0.151–0.243 m/s; dominant frequency, 1.475–3.443 Hz; 2.951–6.885 calculated cycles

**Table 8:** Temporal distribution of the predicted algorithmic state categories.

Category	Duration, s	Share of Functionally Valid Time, %	Share of the Complete Recording, %	95% Block-Bootstrap Interval, %
Good	549.300	74.431	61.033	60.837–87.505
Neutral	127.367	17.258	14.152	7.424–27.514
Bad	61.333	8.311	6.815	3.976–12.707
Uncertain	162.000	—	18.000	13.326–23.296

**Table 9:** Completeness and geometric quality of the manually annotated 5-min test sequence.

Indicator	Value
Analyzed duration	300.0 s
Frame rate	30 Hz
Synchronized temporal samples	9000
Three-dimensional keypoint records	135,000
Missing three-dimensional coordinates	0
High-error reconstructions	36 (0.0267%)
Median reprojection error	2.381 px
Mean reprojection error	3.112 px
Points supported by three cameras	48.45%
Points reconstructed from the best camera pair	51.55%

**Table 10:** Class-wise state-recognition performance and comparison of state durations.

State	Precision, %	Recall, %	F1, %	Ground Truth, s	Algorithm, s
Standing	94.17	90.56	92.33	148.00	142.33
Walking	74.87	88.06	80.93	60.00	70.57
Lying	100.00	94.28	97.05	92.00	86.73

**Table 11:** Limited manual temporal validation on the second 5-min synthetic sequence.

Reference Target	Ref./Pred.	Precision/Recall / F1, %	Temporal Results
Global-state transitions	15 / 16	87.5 / 93.3 / 90.3	TP = 14, FP = 2, FN = 1; onset MAE, 0.926 s; median absolute onset error, 0.850 s; mean state-interval IoU, 0.895; offset MAE, 0.875 s
Prolonged-immobility intervals	2/2	100/100/100	TP = 2, FP = 0, FN = 0; onset MAE, 1.317 s; mean confirmation latency, 10.033 s; offset MAE, 0.750 s; mean IoU, 0.881; false alarms, 0 h^−1^

**Table 12:** Restricted validation of the posture-derived Good/Neutral functional categories on the second 5-min synthetic sequence.

Reference	Predicted Good	Predicted Neutral	Uncertain	Precision, %	Recall, %	F1, %
Good	7846	31	13	98.80	99.61	99.20
Neutral	95	1012	3	97.03	91.42	94.14

**Table 13:** Sensitivity of the state-classification and quality-gate outputs and fall-threshold output stability on the second 5-min synthetic evaluation sequence.

Parameter Group	Change	State Accuracy, %	Macro-F1, %	Coverage, %	Observed Output
Baseline	0	97.40	97.77	99.83	3 POOR frames
State-membership ramps	±10%,±20%	96.97–97.57	97.30–98.02	99.83	Stable state output
Quality gate	−20%	—	—	99.85	0 POOR; 2 prolonged immobility; 1 convulsion-like
Quality gate	−10%	—	—	99.85	0 POOR; 2 prolonged immobility; 1 convulsion-like
Quality gate	+10%	—	—	95.19	423 POOR; 1 prolonged immobility; 0 convulsion-like
Quality gate	+20%	—	—	90.35	912 POOR; 0 prolonged immobility; 0 convulsion-like
Fall thresholds	±10%,±20%	—	—	—	No change in fall output

**Table 14:** Controlled ablation results for the principal components of the three-dimensional and temporal-analysis pipeline.

Component	Comparison	Quantitative Result	Interpretation
Confidence weighting	Weighted vs. unweighted triangulation	MPJPE: 31.8645 vs. 31.8639 mm	No measurable effect under the clean synthetic acquisition conditions
Filtering	Raw/cleaned/smoothed streams	MPJPE: 31.87/34.17/71.75 mm; P95: 88.77/92.78/196.40 mm	Temporal smoothing targets trajectory stability and short-term jitter suppression; the larger frame-aligned error reflects temporal lag rather than improved instantaneous positional accuracy
Quality gate	Enabled vs. disabled	Coverage: 99.83% vs. 99.85%; accuracy: 97.40% vs. 97.40%; macro-F1: 97.77% vs. 97.77%	Minimal effect on this clean sequence because only three baseline frames were classified as POOR
Temporal confirmation	Enabled vs. disabled	Accuracy: 97.40% vs. 98.77%; macro-F1: 97.77% vs. 99.00%; coverage: 99.83% vs. 99.95%; state changes: 17 vs. 20; fall activations: 0 vs. 1	Confirmation reduces temporal fragmentation and downstream false activation at the cost of a small frame-level delay

## Data Availability

The data presented in this study are available from the corresponding author upon reasonable request.
